# The Impact of the Universal Infant Free School Meal Policy on Dietary Quality in English and Scottish Primary School Children: Evaluation of a Natural Experiment

**DOI:** 10.3390/nu14081602

**Published:** 2022-04-12

**Authors:** Jennie C. Parnham, Kiara Chang, Christopher Millett, Anthony A. Laverty, Stephanie von Hinke, Jonathan Pearson-Stuttard, Frank de Vocht, Martin White, Eszter P. Vamos

**Affiliations:** 1 Public Health Policy Evaluation Unit, School of Public Health, Imperial College London, London W6 8RP, UK; chu-mei.chang@imperial.ac.uk (K.C.); c.millett@imperial.ac.uk (C.M.); a.laverty@imperial.ac.uk (A.A.L.); e.vamos@imperial.ac.uk (E.P.V.); 2 Erasmus School of Economics, Erasmus University Rotterdam, 3062 PA Rotterdam, The Netherlands; s.vonhinke@bristol.ac.uk; 3 School of Economics, University of Bristol, Priory Road Complex, Bristol BS8 1TU, UK; 4 Department of Epidemiology and Biostatistics, School of Public Health, Imperial College London, London SW7 2AZ, UK; j.pearson-stuttard@imperial.ac.uk; 5 Northumbria Healthcare NHS Foundation Trust, Newcastle upon-Tyne NE27 0QJ, UK; 6 Health Analytics, Lane Clark & Peacock LLP, London W1U 1DQ, UK; 7 Population Health Sciences, Bristol Medical School, University of Bristol, Bristol BS8 1QU, UK; frank.devocht@bristol.ac.uk; 8 MRC Epidemiology Unit, University of Cambridge, Cambridge CB2 0QQ, UK; martin.white@mrc-epid.cam.ac.uk

**Keywords:** school lunch, nutrition, children, universal free school meals, policy evaluation

## Abstract

The Universal Infant Free School Meal (UIFSM) policy was introduced in September 2014 in England and January 2015 in Scotland and offered all infant schoolchildren (ages 4–7 years) a free school lunch, regardless of income. Yet, impacts of UIFSM on dietary intakes or social inequalities are not known. A difference-in-differences study using the National Diet and Nutrition Survey assessed pooled pre-UIFSM (2010–2014) and post-UIFSM (2014–2017) dietary data. English or Scottish infant schoolchildren (4–7 years; n = 458) were the intervention group, with junior schoolchildren (8–11 years; n = 401) as controls. We found that implementation of UIFSM led to an increase in infant schoolchildren having a school meal. Impacts on key food groups such as fruit and vegetables or sweetened beverages were not seen. However, there was evidence that the UIFSM policy lowered consumption of foods associated with packed lunches, such as crisps, and some nutrients, such as total fat and sodium. Policy impacts differed by income group, with larger effect sizes in low-income children. In conclusion, evaluation of UIFSM demonstrated some improvements in dietary quality but the findings suggest school meal quality needs to be improved to fully realise the benefits of UIFSM.

## 1. Introduction

Currently in the UK, evidence suggests that school meals are healthier than packed lunches [1,2,3,4,5,6,7]. This is partly attributable to School Food Standards introduced between 2006 and 2015 to regulate school meal quality, which only 1% of packed lunches meet [8]. Despite this, school meal uptake in 2013/2014 was low among infant schoolchildren (38%) [9], meaning the majority of schoolchildren ate a lunch which was of poorer dietary quality. This is important as children consume one-third of their diet at school, and therefore their lunch substantially contributes to the quality of their overall dietary intake [1,6,10]. Dietary habits established in early life have been shown to track into adulthood [11], so encouraging healthy eating in school may contribute to long-term improved dietary intake and health outcomes. Furthermore, schools represent a large subsection of the population, including all children but also teachers, staff, and family. Therefore, the school food environment represents a potentially efficient and equitable way of impacting children’s dietary intake on a large scale.

In the UK, free school meals (FSM) are provided through a means-tested scheme to primary and secondary schoolchildren from low-income families who receive specific income-related state benefits (see Table S1 for details). This represents 17.3% of the school population [12]. However, this scheme does not reach all of the 31% of UK children estimated to be in relative poverty [13,14], leaving them vulnerable to food insecurity, including a poor-quality packed lunch [4,7]. In September 2014, the introduction of the Universal Infant Free School Meal (UIFSM) scheme made school meals free to all English infant schoolchildren (ages 4–7 years), regardless of their income. Scotland implemented this policy in January 2015. The stated aims of the programme were to ensure children had access to one healthy meal, develop long-term healthy eating habits, improve educational attainment, help families with the cost of living and remove disincentives to work for caregivers [15].

A pilot study implemented UIFSM in primary schools in two local authorities in England between 2009 and 2011 and compared outcomes to those in control local authorities [16]. The study reported a 28% increase in uptake of school meals and a shift away from cold and snack foods (crisps, sandwiches and fruit) to hot cooked foods (potatoes, pasta and vegetables). However, this pilot did not examine the effect on nutrient intakes. A before–after study of two infant schools in Northumberland (n = 196, 2008–2017) showed a lower intake of sugar after UIFSM [17].

Since the implementation of the UIFSM scheme, school meal uptake rose to 80% in infant schoolchildren in 2015/2016 [15]. This increase in uptake may have resulted in an improved dietary intake of infant schoolchildren, especially for low-income children who were not eligible for means-tested FSM. However, the effect of UIFSM on average dietary intake at a national level or what the impacts were for children from different socioeconomic backgrounds are not known.

The primary aim of this study is to evaluate the impact of the UIFSM policy on the dietary quality of lunchtime intake in infant schoolchildren using detailed, nationally representative dietary data. The secondary aim is to investigate whether the policy impact differed by household income.

## 2. Materials and Methods

### 2.1. Study Design

We conducted a natural experimental evaluation using a difference-in-differences (DID) approach to evaluate the impact of the UIFSM policy on dietary quality [18]. We estimated policy impacts by comparing average changes in consumption of food items and nutrients at two time points (pre-UIFSM (2010–2014) and post-UIFSM (2014–2017)) between intervention (infant schoolchildren, ages 4–7 years) and control (junior schoolchildren, ages 8–11 years) groups. Junior schoolchildren were used as a control group as they are closest in age, and are in the same primary school environment, but were not eligible for UIFSM. Therefore, this study tests the impact of FSM becoming universal compared to the previous, means-tested system.

### 2.2. Data Source

The National Diet and Nutrition Survey (NDNS) [19] is a rolling programme of cross-sectional surveys which provides a representative snapshot of dietary intake in the UK among those aged 1.5 years and over. A multi-stage random sampling method was used to select households from a list of postcodes; further details are published elsewhere [20]. Data from annual surveys in the period 2010–2017 were analysed. Dietary data in the NDNS were measured through a validated three- or four-day food diary, which recorded the location, time and quantity of all food and drink consumed. Diaries were completed by a guardian on behalf of the participants. Sociodemographic variables were collected through an interview-assisted survey.

### 2.3. Study Participants

All children (ages 4–11 years) from England or Scotland who recorded a lunchtime dietary intake on a school day were included. From the initial sample (n = 1127), participants were excluded if they were not at school during lunch on any of their recorded days (22%, n = 244), their school did not provide food (2%, n = 28) or they did not record their ethnicity (<1%, n = 1), leaving an analytic sample of 854 participants (intervention n = 435; control n = 401).

### 2.4. Exposure Variables

The UIFSM policy was the exposure in the analysis. Due to the small samples in the intervention and control groups, the data were pooled in two time periods. Participants were defined as being in the pre-UIFSM time period if their dietary data were recorded before September 2014 for English participants or January 2015 for Scottish participants, otherwise they were defined as being in the post-UIFSM time period. Small sample sizes also meant we could not exploit differences in UIFSM introduction between England and Scotland. Indicators of intervention time period (0 = pre-UIFSM, 1 = post-UIFSM) and intervention group (0 = control, 1 = intervention) were created.

### 2.5. Outcome Variables

Only food and drink items recorded between 12:00 and 14:00 p.m. on a weekday at a school premise were included in the lunchtime variables. Food and nutrient intakes at lunch were averaged to one ‘standard’ lunchtime, where multiple days were recorded (1 school day [n = 111, 13%], 2 school days [n = 345, 40%], 3 school days [n = 229, 27%], and 4 school days [n = 169, 20%]).

In total, 22 food group variables and 20 nutrient variables were available to evaluate changes in dietary quality. Food group variables were informed by the School Food Standards and by previous literature. Food groups (in g/lunch) included wholemeal foods, starchy food (not cooked in oil), starchy foods (cooked in oil), biscuits, crisps, puddings, sweets, sugar-sweetened beverages (SSB), dairy, fruit and vegetables and foods high in salt, fat or sugar, milk, yoghurt, cheese, high-protein foods, baked beans, fruit juice, fruit, vegetables, and water. Foods were defined as being high in either salt, fat or sugar using the UK food labelling guidance [21]. See Table S2 for detailed descriptions of food groups. Nutrient variables included energy (Kcal/lunch), total fat (g/lunch), saturated fat (g/lunch), carbohydrate (g/lunch), non-milk extrinsic sugar (NMES) (g/lunch), protein (g/lunch), sodium (mg/lunch), fibre (g/lunch), calcium (mg/lunch), iron (mg/lunch), zinc (mg/lunch), potassium (mg/lunch), folate (ug/lunch), vitamin C (mg/lunch), vitamin A (ug/lunch), total fat (% energy lunch), saturated fat (% energy lunch), carbohydrate (% energy lunch), NMES (% energy lunch) and protein (% energy lunch). Dietary supplements were excluded from all variables.

Total daily nutrient intakes were additionally analysed to assess if changes in lunchtime nutrient intakes were compensated later in the day. Total daily nutrient intakes were calculated as the sum of all food consumed on the day the child attended school, averaged to one school day if multiple school days were recorded.

### 2.6. Covariates

Covariates included age (years); sex, ethnicity (White or Ethnic Minority groups); equivalised household income (£); index of multiple deprivation (IMD) (quintiles); geographical region (North England, Central England, South England, and Scotland) and total lunchtime intake (g/lunch). IMD is an area-based composite measure of relative deprivation in the UK [22]. All study covariates were complete except for household income, which was missing for 11% of the sample (n = 94). Missing income was imputed using the Classification and Regression Trees (CART) method with 10 iterations specified [23]. Income was subsequently adjusted for inflation using consumer price indices, with 2017 as the reference year.

### 2.7. Statistical Analysis

Bivariate statistical tests compared sample characteristics between intervention and control groups, separately for pre- and post-UIFSM periods. χ^2^ and *t*-tests were used for categorical and continuous variables, respectively.

A DID framework was employed to evaluate the impact of the UIFSM policy on dietary intakes. This was modelled using a regression-based approach that included intervention period, intervention group, and an interaction between them. The interaction term is the DID estimator that measures the effects of the policy by comparing average changes in the outcome from pre- to post-UIFSM period between intervention and control groups. The control group represents the best estimate of the dietary intake in the intervention group in absence of the policy. We assumed that changes in dietary intakes over time in the intervention group would have followed a parallel trend to that of the control group in the absence of the UIFSM policy. Small annual sample sizes precluded an analysis with multiple time points pre- and post-UIFSM.

Food group outcomes were found to be heavily right skewed, as not all food groups were commonly eaten by all schoolchildren. Therefore, two-part models were used to analyse the effect of UIFSM on intake of food groups. In the first stage, linear probability models were used to assess changes in the probability of consuming a food group, comparing those who had any amount of a food group to those who consumed none. In the second stage, linear regression models were used to examine changes in the average portion size of a food group, including only participants who had consumed some of the food group (intake over 0 g). Nutrient outcomes were normally distributed, and therefore linear regression models were used to assess the impact of UIFSM on nutrient intake. Covariates were included in three models: Model 1 was unadjusted, Model 2 was adjusted for sociodemographic covariates and Model 3 was adjusted for total lunchtime intake (g) (see Table S3 for further details). The analyses were further stratified by income tertile (low, medium, and high) to investigate whether low-income children were differentially impacted compared to higher-income children.

All models were adjusted by inverse probability weights (IPW) to ensure study covariates were well-balanced across all four groups (Pre-Intervention, Pre-Control, Post-Intervention and Post-Control) [24]. The IPW were computed as the inverse of the predicted probability of being in the Pre-Intervention group using a multinomial regression including sex, ethnicity, region, household income, IMD, socioeconomic status, and survey weight variable. Sensitivity analyses were conducted to compare findings with and without IPW adjustment.

Further sensitivity analyses were conducted to assess potential bias due to dietary mis-reporting using the Goldberg method, adapted for children [25,26]. Unreliable energy reporters were identified by comparing a participant’s estimated energy requirements to their reported energy intake. The analyses were repeated excluding participants identified as possible unreliable energy reporters (n = 44, 5%) to assess if findings were robust to reporting bias. 

All data management and analyses were conducted in R (version 4.0.2, PBC, Boston, MA, USA).

## 3. Results

In total, 854 participants were included in this study, 520 in the pre-UIFSM period and 334 in the post-UIFSM period. Take up of school meals pre-UIFSM was comparable for intervention and control groups (47.4% vs. 49.6%, respectively). After UIFSM, the proportion of the intervention group consuming a school meal was significantly higher (80.5%) than the control (48.8%). There were no significant differences in the distribution of sex or ethnicity either between groups or across time periods (Table 1). Additionally, there were no significant differences in income, IMD and region in the post-UIFSM period between the groups. However, in the pre-UIFSM period, the intervention group was more likely to have a higher household income and come from the south of England than the control group. Application of IPW balanced all sample characteristics between intervention groups and periods (see Table S4).

### 3.1. Policy Impact on Lunchtime Food Consumption Patterns

**Figure 1:**
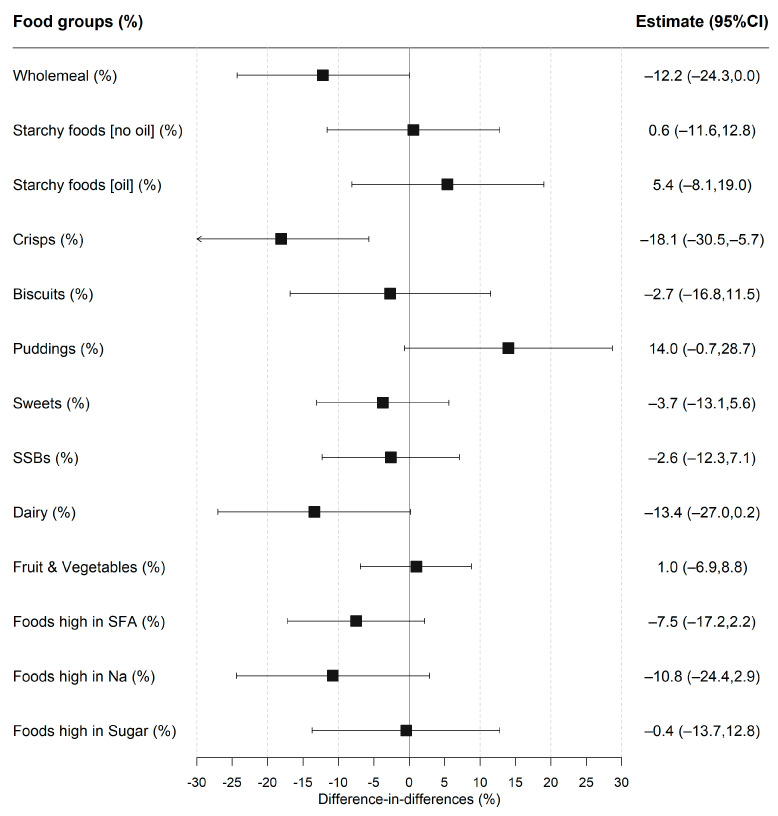
Difference-in-differences estimates of UIFSM policy impact on the likelihood of consuming a food group. Note: SSBs—sugar-sweetened beverages, SFA—saturated fats, and Na—sodium. Linear probability regression adjusted for age, sex, ethnicity, household size, region, household income, IMD and total lunchtime intake. Pre/Post prevalence of consuming food groups presented in Table S3. Coefficients represent percentage point change in likelihood of consuming a food group.

The proportion of children consuming a food group was analysed in the first stage of the two-part model (Figure 1 and Table S3). The foods that were most frequently consumed at lunchtime before the policy included white starchy foods (75.4% vs. 71.3% for intervention and control groups, respectively), fruit and vegetables (95.0% vs. 91.1%) and foods high in salt (69.8% vs. 70.9%), fat (87.9% vs. 86.5%), and sugar (66.2% vs. 71.3%). After the policy had been implemented, there was a decrease in the proportion of the intervention group who ate wholemeal products (−13.9%; 95% CI −21.6, −6.3) and crisps (−11.0%; −18.3, −3.7) but an increase in consuming puddings (16.0%; 5.9, 26.2), with no evidence of a significant change for any food group in the control group. The DID model estimated the policy impact; the estimates remained consistent when accounting for sociodemographic covariates and total lunchtime intake (Table S3). The fully-adjusted results (see Figure 1) showed that the intervention group was less likely to consume crisps after implementation of UIFSM (−18.1%; −30.5, −5.7), with weak evidence of reduction in consumption of wholemeal (−12.2%; −24.3, −0.1; *p* = 0.05) and dairy products (−13.4%; −27.0, 0.2, *p* = 0.05). There was also some evidence that the intervention group was more likely to consume puddings after UIFSM (14.0%; −0.7, 28.7; *p* = 0.06).

**Figure 2:**
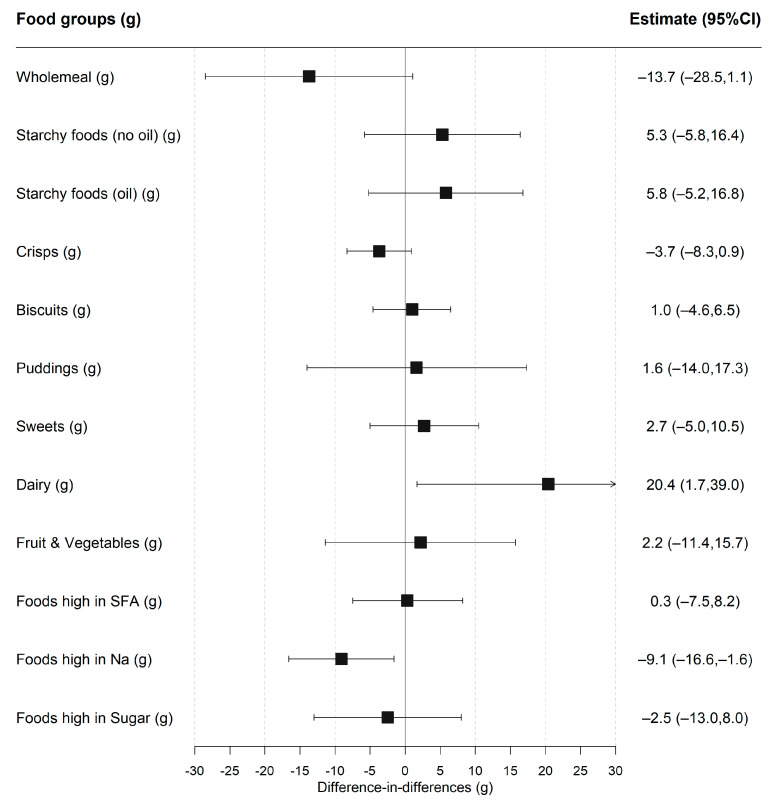
Difference-in-differences estimates of UIFSM policy impact on food groups consumed (g), conditional on consumption. Note: SFA—saturated fats; Na—sodium. Linear regression adjusted for age, sex, ethnicity, household size, region, household income, IMD and total lunchtime intake. Coefficients compare the amount of a food group consumed, conditional on consumption (>0 g). Pre/Post mean intakes of food groups presented in Table S5.

The average portion size of a food group consumed was compared among participants who ate some of that food group in the second stage of the two-part model (Figure 2 and Table S5). The average portion size for white starchy foods (not cooked in oil), sugar sweetened beverages and foods high in sodium decreased after UIFSM for the intervention but not the control group. However, there was only strong evidence for an increase in the amount of dairy consumed (20.4 g; 1.7, 39.0) and a reduction in the amount of foods high in sodium consumed (−9.1 g; −16.6, −1.6), after adjustment for confounders. There was no evidence that the total amount of food consumed at lunch was impacted by UIFSM.

### 3.2. Policy Impact on Lunchtime Nutrient Intakes

**Figure 3:**
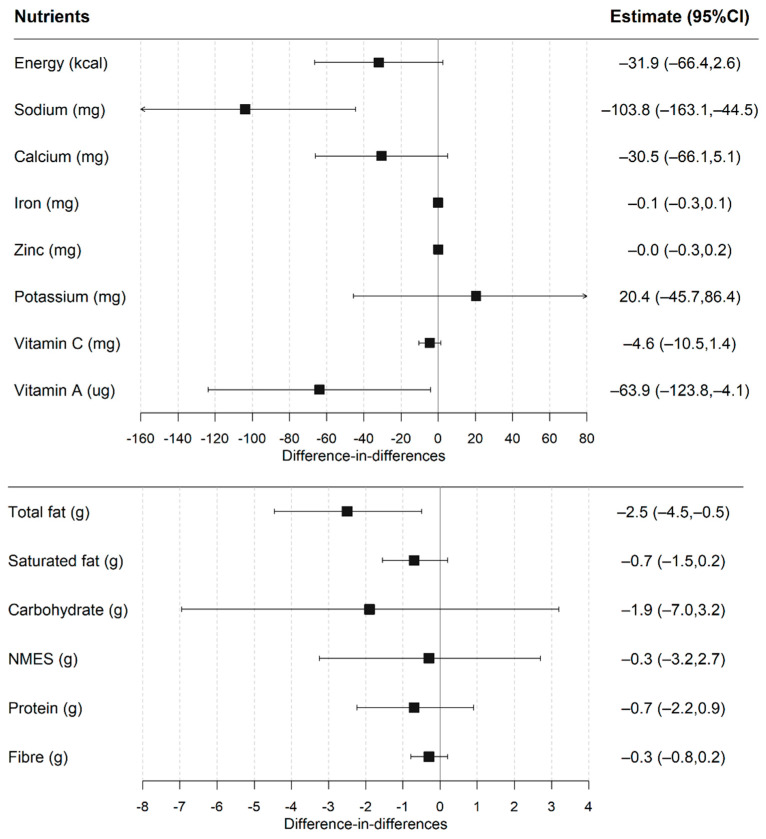
Difference-in-differences estimates of UIFSM policy impact on lunchtime nutrient intakes. Note: NMES—non-milk extrinsic sugar. Linear regression adjusted for age, sex, ethnicity, household size, region, household income, IMD and total lunchtime intake. Pre/Post mean intakes of nutrients presented in Table S6.

The post-UIFSM sodium intakes were significantly lower in the intervention group compared to the control (Figure 3 and Table S6). Estimates of UIFSM impacts on lunchtime nutrient intake were consistent after adjusting for sociodemographic covariates and total lunchtime intake. The fully-adjusted DID model showed that UIFSM resulted in lower total fat (−2.5 g; −4.5, −0.5), sodium (−103.8 mg; −163.1, −44.5) and vitamin A (−63 ug; −123.8, −4.1) intakes. There was weak evidence that UIFSM impacted energy (−31.9 kcal; −66.4, 2.6; *p* = 0.07) and calcium intakes (−30.5 mg; −66.1, 5.1; *p* = 0.09). A comparable effect was also observed when total fat was analysed relative to energy intake, which accounts for differences in total energy intake between person (Table S6).

### 3.3. Sensitivity Analyses

Sensitivity analyses showed results were broadly similar with and without IPW adjustment (Tables S7 and S8). Additionally, neither the direction of effect nor significance of the results were affected by removing participants identified as possible energy mis-reporters (see Tables S9 and S10).

### 3.4. Policy Impact on Daily Nutrient Intakes

Total mean sodium levels across the school day were lower in the intervention group post-UIFSM; however, this effect was not observed after accounting for confounders in the DID model (−83.1 mg/day; −215.1, 49.0) (Table S11). After adjustment, there was some evidence that the UIFSM policy was associated with a reduction in fat as a proportion of total energy across the day (−1.5% fat of total energy; −3.01, −0.03).

### 3.5. Differences between Income Groups

**Figure 4:**
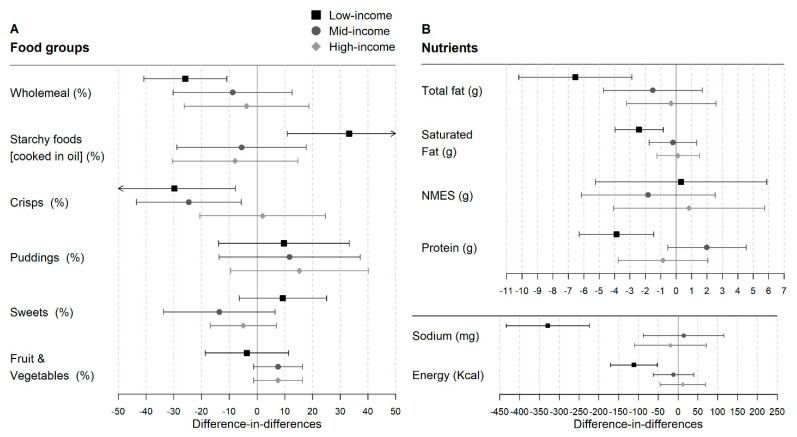
Difference-in-differences estimates of UIFSM policy impact on selected study outcomes, stratified by income level. (**A**) Food group outcomes. Linear probability regression adjusted for age, sex, ethnicity, household size, region, household income, IMD and total lunchtime intake. Coefficients represent percentage point change in likelihood of consuming a food group. Pre/Post intakes presented in Table S12. (**B**) Nutrient outcomes. Linear regression adjusted for age, sex, ethnicity, household size, region, household income, IMD and total lunchtime intake. Pre/Post mean intakes of nutrients presented in Table S13. NMES—non-milk extrinsic sugar.

Lunchtime intakes of food groups and nutrients were broadly similar in high-, middle- and low-income intervention groups before implementation of UIFSM (Tables S12 and S13). Except for wholemeal and vegetable products, which were less frequently consumed in the low- compared to the high-income intervention group. Following implementation of UIFSM, there was a significant reduction in the likelihood of consuming crisps (−32.8%; −54.9, −10.6), wholemeal products (−25.7%; −40.5, −10.8) and foods high in saturated fat (−26.5%; −42.6, −10.5) (Figure 4A) in the low-income but not in the middle- or high-income groups. UIFSM was also associated with a significant increase in the proportion of the low-income group eating starchy foods cooked in oil (31.7%; 9.6, 53.8), milk (18.3%; 3.7, 33.0) and water (22.1%; −1.3, 45.5), but not in the middle- or high-income groups. For the middle-income children, there was weak evidence of an increased consumption of puddings (25.1%; 0.3, 49.8).

When nutrients were analysed, a decrease was observed in total fat (−7.1 g; −10.7, −3.4) and sodium (−331.0 mg; −434.8, −227.1) as well as energy (−122.7 kcal; −181.7, −63.6), protein (−3.9 g; −6.3, −1.5), iron (−0.6 mg; −0.9, −0.3), zinc (−0.5 mg (−0.8, −0.2), and calcium (−99.7 mg; −157.2, −42.2) in the lowest-income group after implementation of UIFSM (Figure 4B and Table S13). However, there was no significant change in the middle- and high-income groups.

## 4. Discussion

### 4.1. Summary of Main Findings

This study evaluated the impact of UIFSM on lunchtime and total daily dietary intake in a representative sample of primary schoolchildren in England and Scotland. Implementation of UIFSM was associated with a 33 percentage point increase in school meal uptake. We found little evidence that UIFSM affected the lunchtime intake of food groups such as fruit and vegetables or SSB. However, there was a reduction in consumption of foods associated with packed lunches, such as crisps, wholemeal and dairy products and an increase in consuming foods associated with school meals, such as puddings. These changes were accompanied by a reduction in total fat, sodium and vitamin A intake at lunch. However, there was no detected change in the amount of sugar consumed. Importantly, the beneficial effect of UIFSM appeared to be greater for low-income children.

Our results show a reduction in the total fat, sodium and vitamin A consumed at lunchtime, demonstrating that universal school-based food interventions can have a measurable impact on children’s lunchtime intake. These nutrients are high in common packed lunch items such as crisps and dairy products, which also lowered after UIFSM. The relative change in vitamin A appears to be concerning; however, the absolute intake remained above reference levels at both lunch (175 µg/lunch) [27] and across the day (500 µg/day) [28] in the post-intervention period. Furthermore, only a difference in fat intake (−1.5% energy; −3.0, −0.0) was observed in the total daily dietary intake, indicating children may compensate for the reduction in sodium at other points in the day. Although this change in fat intake is modest, it has potential to accumulate to a meaningful beneficial impact on child health over time and at a population level.

We found no evidence that UIFSM affected sugar intake at lunchtime or in the total diet. The likelihood of infant schoolchildren consuming sugary foods was unchanged, with over 70% consuming foods high in sugar post-UIFSM. This may indicate that children were switching the source of their sugar intake, for example there is some indication that intake of SSB (before–after difference: −6.3%; CI −13.2, 0.6; *p* = 0.08) and yoghurts (before–after difference: −13.5%; CI −22.9, −4.2; *p* < 0.01) in packed lunches were replaced with puddings in school lunches (before–after difference: 16.0%; CI 5.9, 26.2; *p* < 0.01). The School Food Standards explicitly restrict foods high in sugar, such as sugary snacks and confectionery. Therefore, it was expected that UIFSM would lower sugar intake and it is of concern that intake of these items did not decrease post-UIFSM. Action to remove sugary items from school menus is needed to better enable children to reduce their sugar intake.

Our analyses indicated that UIFSM effects were greater for children from low-income families compared to children from high-income families. The low-income group in this study included children who were either eligible or ineligible for the means-tested FSM prior to introduction of UIFSM. National estimates indicate that the uptake of school meals in means-tested FSM pupils were minimally affected by UIFSM, rising only two percentage points to around 87% [9,12]. The rise in school meal uptake due to reduced stigma for FSM-eligible pupils was lower in national estimates compared to UIFSM pilot studies [29]. Consequently, it is surprising that the middle-income group, who had a larger shift from packed lunches to school meals (43 percentage point increase) than the low-income group (26 percentage point increase), did not see a similar effect in their lunchtime intake. The means-tested FSM eligibility is dependent on receiving social security benefits. As such, it is estimated two out of five children in poverty are ineligible for a means-tested FSM [14]. This is partly due to a rise in in-work poverty for households with children [30]. Children who are low income but ineligible for FSM have been shown to have less to spend on their school lunches than more affluent children and are more likely to take a packed lunch (80%) [31], and therefore are most at risk of taking a poor-quality packed lunch. We were unable to differentiate children who were eligible for a means-tested FSM in the low-income group from those who were not. However, it seems reasonable to hypothesise that the large differences in the low-income group were driven by the children who were not eligible for means-tested FSM. These children likely experienced a large change in their lunchtime intake compared to FSM-eligible children whose lunchtime provision remained similar. In this way, UIFSM has greater potential than the means-tested FSM to address socioeconomic inequalities in diet, by reaching a larger proportion of low-income children.

### 4.2. Relationship to Previous Research

A systematic review of universal free school meal programmes worldwide showed a similar positive impact on school meal uptake [32]. For example, the Community Eligibility Provision (CEP) in the United States provides schools with >40% children from low-income households funding to deliver universal school meals [33]. Regional evaluations on how CEP impacted school meal participation revealed positive results, an 8% increase in Maryland and Pennsylvania [34] and a 4 percentage point increase in Texas [35]. Notably, the impact of UIFSM appears to be greater than the CEP. Furthermore, the 30% increase in participation we estimate is consistent with previously reported figures on the UIFSM [16]. However, in the UK, there have only been two prior studies evaluating the impact of UIFSM on dietary intake, neither of which were nationally representative. A pilot, conducted before UIFSM was implemented, reported a shift from consuming cold foods (crisps and sandwiches) to hot foods (pasta and chips), and no significant change in an aggregated sweet foods category [16]. Similarly, in a before–after study with no control group, Spence et al. [17] observed a lower intake of yoghurts, a higher consumption of cake in one school studied but overall a lower biscuit and sugar intake after UIFSM. Our findings demonstrate a similar pattern in dietary intake to the previous literature. Furthermore, our study adds new data demonstrating how the nutrient profile of lunchtime intakes were altered at a national level, for example lowering lunchtime sodium (−103.8 mg; −163.1, −44.5) and total fat (−2.5 g; −4.5, −0.5). Holford et al. [9] analysed national weight data for English schoolchildren and demonstrated that children (aged 4–5 years) exposed to UIFSM for longer had a small but significant increased likelihood of being a healthier weight (1.2% on average) than less exposed children. Our analyses showed weak evidence for lower energy intake at lunch (−31.9 kcal; −66.4, 2.6; *p =* 0.07), but only a reduction in fat remained in the total daily diet. It is probable that the difference in the sample size between the two studies can explain the difference in results, Holford et al. had over 154,000 first-year schoolchildren, giving their analysis more statistical power to detect small changes.

### 4.3. Strengths and Limitations

This is the first study to evaluate the national impact of the UIFSM on dietary intakes. The nationally representative dataset used has detailed dietary data, permitting us to examine the effect on a wide range of foods and nutrients and narrow dietary intakes to a specific time and location, generalisable to infants in England and Scotland. Additionally, we were able to assess the effect on total nutrient intake across the day, to assess whether intakes were compensated outside school. Moreover, this is the first study to evaluate the impact between income groups, highlighting important socioeconomic differences.

There are, however, some limitations to note. Despite being universal, not all infants chose to take-up the scheme, and as such the results represent the “intention-to-treat” effect of the policy. This implies the estimated policy impacts may thus be diluted by the 20% of infants who took a packed lunch post-UIFSM. Potential limitations include changes in the food environment which occurred over the study period, such as the new School Food Standards being introduced in January 2015. The DID method accounted for this by using junior schoolchildren as the counterfactual; as they are in the same primary school environment, they are affected by the same environmental changes. However, a limitation is that junior schoolchildren have slightly different metabolic requirements. The UIFSM policy has not been implemented in Wales and Northern Ireland, and therefore infant schoolchildren in this setting would have been a more appropriate counterfactual. However, the sample size was too small for them to be used (pre n = 110, post n = 70). Another limitation is the potential bias due to dietary misreporting. However, sensitivity analyses which removed possible energy misreporters showed similar results, the biggest divergence being a reduction of 9.6 mg in sodium (−94.2 mg; −155.0, −33.4). Additionally, there was variation in the number of days recorded between participants, from one to four days, leading to variation in the level of measurement error in the outcome across the sample. This may lead to increased standard errors but not bias in the estimates.

The NDNS did not record whether a child attended a state or independent school. Therefore, any independently educated children in the sample might not have been exposed to the UIFSM scheme, which is only available in state schools. National statistics estimate 0.6% of primary-school aged children attend independent schools [12], and therefore the impact of bias from this source of misclassification is likely to be small. Moreover, it is unlikely that the prevalence of independent schools would vary pre- and post-UIFSM. Additionally, there is a chance of an overspill effect from junior schoolchildren who were exposed to UIFSM in infant school; but as the number is small (n = 27), this is also unlikely to impact the findings. Finally, this study is limited by a relatively small sample size; to counteract this, we pooled the data across years to maximise the sample size but there are wide confidence intervals surrounding our estimates. As a result, although the DID design enabled inferences about the programme’s effectiveness, estimates of its effect size are somewhat imprecise.

### 4.4. Implications for Policy and Research

Our study reports some positive effects of the UIFSM on dietary intakes, but these were not consistent across all outcomes. We hypothesise that this was due to school meals not consistently meeting School Food Standards. For example, consumption of restricted foods, such as foods high in sugar, did not lower; meanwhile, recommended foods, such as fruit and vegetables, did not increase. These findings put a renewed focus on the content of school meals, which although in most cases are preferable to packed lunches, are not always optimal [1]. Variation in the food environment between schools is a major contributing factor to the nutritional quality of school meals. The School Food Standards are mandatory but are not monitored in England. As such, school food quality is determined more by school leadership than central governance. The amount given to schools for UIFSM has only risen once in seven years (from £2.30/meal to £2.34/meal in 2020). Some school leaders suggest this is insufficient and puts increased strain on already limited educational resources [15]. Without adequate funding, support for UIFSM by senior leadership is inconsistent [15,36]. Budget constraints in combination with lack of incentives for complying with School Food Standards are reported to contribute to non-compliance in schools [37]. In a sample of London primary schools, only 43% had a School Food Standards-compliant food policy [38]. As such, the capability of UIFSM to improve dietary intake is limited by a school’s resources to improve their food environment, which may explain why the UIFSM policy has not yet achieved its potential. To help identify areas for improvement, future research should evaluate the compliance of school meals to School Food Standards across a range of school types and deprivation levels.

## 5. Conclusions

This study demonstrated that UIFSM was associated with some improvements in dietary quality, such as lower total fat and salt intake. However, changes were not seen in outcome such as sugar. Additionally, we present evidence that UIFSM may reduce socioeconomic inequalities. Improvements to the school food environment are needed to ensure that children have access to healthy food and the benefits of UIFSM are maximised.

## Figures and Tables

**Table 1:** Characteristics of schoolchildren (n = 854) in England and Scotland before and after implementation of the UIFSM policy.

		Pre-UIFSM (2010–2014) ^1^			Post-UIFSM (2014–2017)		
Variable		Intervention Group: Infants (n = 281)	Control Group: Juniors (n = 239)	*p* Value ^2^	Intervention Group: Infants (n = 172)	Control Group: Juniors (n = 162)	*p* Value ^3^
**Age**	Mean (SD)	5.6 (1.0)	9.1 (1.0)	<0.001 ^b^	5.7 (1.0)	9.4 (1.0)	<0.001 ^b^
**Sex**	n (%)			0.28 ^a^			0.45 ^a^
Female		137 (49.9)	107 (44.3)		88 (50.0)	72 (45.4)	
**Ethnicity**	n (%)			0.98 ^a^			0.70 ^a^
Ethnic minorities		52 (20.2)	43 (20.1)		30 (20.0)	27 (18.1)	
**Household income (£)**	Mean (SD)	30,648.3 (19,539.5)	26,601.9 (18,800.4)	0.03 ^b^	31,479.6 (20,798.4)	28,976.4 (18,465.9)	0.28 ^a^
**IMD (quintiles)**	n (%)			0.07 ^a^			0.44 ^a^
Least deprived		62 (19.1)	42 (19.4)		40 (20.8)	42 (27.6)	
2		49 (17.4)	41 (14.9)		29 (17.7)	29 (18.3)	
3		60 (23.2)	44 (16.5)		33 (21.0)	27 (13.5)	
4		64 (24.0)	52 (21.2)		34 (19.1)	32 (20.9)	
Most deprived		46 (16.3)	60 (28.1)		36 (21.4)	32 (19.7)	
**Region**	n (%)			0.03 ^a^			0.16 ^a^
England: North		57 (21.9)	62 (31.3)		44 (24.0)	37 (20.5)	
England: Central		44 (13.4)	42 (17.8)		28 (16.7)	25 (14.1)	
England: South		126 (55.3)	83 (40.9)		87 (51.8)	77 (49.1)	
Scotland		54 (9.5)	52 (10.0)		13 (7.6)	23 (16.3)	
**School lunch type**	n (%)			0.68 ^a^			<0.001 ^a^
School meal		139 (47.4)	121 (49.6)		141 (80.5)	78 (48.8)	
Packed lunch		142 (52.6)	118 (50.4)		31 (19.5)	84 (51.2)	

^1^ Threshold is September 2014 for English participants and January 2015 for Scottish participants; ^2^ pre-UIFSM intervention vs. pre-UIFSM control; ^3^ post-UIFSM intervention vs. post-UIFSM control; ^a^ Chi-square test (adjusted for survey weights); ^b^ *t*-test (adjusted for survey weights). Note: UIFSM—Universal Infant Free School Meal; SD—standard deviation; IMD—index of multiple deprivation.

## Data Availability

The dataset analysed in this study is available in the UK data service (SN: 6533). Accessed from http://doi.org/10.5255/UKDA-SN-6533-17 (accessed on 2 February 2020).

## Supplementary Materials

The following supporting information can be downloaded at: https://www.mdpi.com/article/10.3390/nu14081602/s1. Table S1. The eligibility criteria of the UK’s means-tested Free School Meal programme between 2004 and 2022. Table S2. Description of food group variables; Table S3. Prevalence of food-group consumption in schoolchildren before and after UIFSM implementation and estimates of UIFSM policy impact; Table S4. Characteristics of schoolchildren (n = 854) in England and Scotland before and after the UIFSM policy with IPW applied; Table S5. Mean lunchtime food group consumption, conditional on consumption, in schoolchildren before and after UIFSM implementation and estimates of UIFSM policy impact; Table S6. Mean lunchtime nutrient intakes in schoolchildren before and after UIFSM implementation and estimates of UIFSM policy impact; Table S7. Difference-in-differences estimates for the impact of UIFSM on food-group outcomes using different specifications of IPW and level of adjustment; Table S8. Difference in difference estimates for the impact of UIFSM on nutrient outcomes using different specifications of IPW and level of adjustment; Table S9. Difference-in-differences estimates for the impact of UIFSM on food-group outcomes after ex-cluding unreliable energy reporters; Table S10. Difference in difference estimates for the impact of UIFSM on nutrient outcomes after excluding unreliable energy reporters; Table S11. Mean daily nutrient intakes in schoolchildren, on a school day, and estimates of UIFSM policy impact; Table S12. Prevalence of food-group consumption in schoolchildren and estimates of UIFSM policy impact stratified by income; Table S13. Mean lunchtime nutrient intakes in schoolchildren and estimates of UIFSM policy impact strati-fied by income.
