# Supplementary material for: The Impact of the Universal Infant Free School Meal Policy on Dietary Quality in English and Scottish Primary School Children: Evaluation of a Natural Experiment
Source: Nutrients. 2022 Apr 12;14(8):1602. doi: 10.3390/nu14081602 (PMC9029848; doi:10.3390/nu14081602)
Supplement: Supplementary file 1 [file nutrients-14-01602-s001.zip › nutrients-1594088-supplementary.pdf]

Supplementary files

Citation: Parnham, J.C.; Chang, K.; Millett, C.; Laverty, A.A.; von Hinke, S.; Pearson-Stuttard, J.; de Vocht, F.; White, M.; Vamos, E.P. The Impact of the Universal Infant Free School Meal Policy on Dietary Quality in English and Scottish Primary School Children: Evaluation of a Natural Experiment. *Nutrients* **2022**

List of tables

Table S1 The eligibility criteria of the UK's means-tested Free School Meal programme between 2004-2022

Table S2 Description of food group variables

Table S3 Prevalence of food-group consumption in schoolchildren before and after UIFSM implementation and estimates of UIFSM policy impact

Table S4 Characteristics of schoolchildren (n=854) in England and Scotland before and after the UIFSM policy with IPW applied.

Table S5 Mean lunchtime food group consumption, conditional on consumption, in schoolchildren before and after UIFSM implementation and estimates of UIFSM policy impact

Table S6 Mean lunchtime nutrient intakes in schoolchildren before and after UIFSM implementation and estimates of UIFSM policy impact

Table S7 Difference-in-differences estimates for the impact of UIFSM on food-group outcomes using different specifications of IPW and level of adjustment

Table S8 Difference in difference estimates for the impact of UIFSM on nutrient outcomes using different specifications of IPW and level of adjustment

Table S9 Difference-in-differences estimates for the impact of UIFSM on food-group outcomes after ex-cluding unreliable energy reporters

Table S10 Difference in difference estimates for the impact of UIFSM on nutrient outcomes after excluding unreliable energy reporters

Table S11 Mean daily nutrient intakes in schoolchildren, on a school day, and estimates of UIFSM policy impact

Table S12 Prevalence of food-group consumption in schoolchildren and estimates of UIFSM policy impact stratified by income

Table S13 Mean lunchtime nutrient intakes in schoolchildren and estimates of UIFSM policy impact strati-fied by income

Table S1. The eligibility criteria of the UK's means-tested Free School Meal programme between 2004-2022.

Year	Benefit	Threshold
2004	Income Support	
	Income-based Jobseeker's Allowance	
	Support under Part VI of the Immigration and Asylum Act 1999	
	Child Tax Credit threshold	£13,480.
2005	+ Guaranteed element of State Pension Credit	
	Child Tax Credit threshold	£13,910
2006	Child Tax Credit threshold	£14,155
2007	+ Income-related employment and support allowance	
	Child Tax Credit threshold	£14,495
2008	Child Tax Credit threshold	£15,575
2009	+ Working tax credit four-week-run-on	
	Child Tax Credit threshold	£16,040
2010	Child Tax Credit threshold	£16,190
2011	FIXED Child Tax Credit threshold	£16,190
2012	+ Universal Credit	
2013-2017	Universal Credit	If receipt after 29 April 2013
2018- Present	Universal Credit	£7,400 per annum.

Source: Freedom of Information request to the Department for Education.

Table S2. Description of food group variables.

Variable	Description
Wholemeal	Any wholemeal starchy product, including wholemeal bread, wholemeal pasta and brown rice
Starchy foods (no oil)	Non-wholemeal starchy products which are not cooked in oil, including white bread, white pasta, white rice and jacket potatoes
Starchy foods (cooked in oil)	Non-wholemeal starchy products which are cooked in oil, including chips and wedges
Biscuit	Any manufactured/retail or homemade biscuit
Crisps	Any crisps or savoury snacks (Includes all potato and cereal based snacks, popcorn (not sweet), twiglets, pretzels, pork scratchings)
Puddings	Buns, cakes, pastries, fruit pies, cereal based milk puddings, sponge puddings, and other cereal based puddings (manufactured/homemade)
Confectionery	Any sugar or chocolate confectionery
Sugar-sweetened beverages	Soft drinks not low calorie
Dairy	Total dairy milk, yogurt, or cheese including from composite dishes.
Fruit and vegetables	Total fruit and vegetables, including from composite dishes. Includes canned but does not include dried fruit.
Milk	Any dairy milk including from composite dishes.
Yoghurt	Any yogurt including from composite dishes.
Cheese	Any cheese including from composite dishes.
Meat, fish and eggs	Any meat, fish, or eggs including from composite dishes.
Baked beans	Any beans, including from composite dishes.
Fruit juice	All fruit juice and smoothies
Fruit	All fruit, including from composite dishes. Includes canned but does not include dried fruit.
Vegetables	All vegetables, including from composite dishes. Includes canned.
Water	Tap or still or carbonated bottled water, no flavouring
High in saturated fat	Any dish with >5g SFA per 100g of food
High in sodium	Any dish with >1.5g salt per 100g of food
High in sugar	Any dish with> 22.5g sugar per 100g of food

Table S3. Prevalence of food-group consumption in schoolchildren before and after UIFSM implementation and estimates of UIFSM policy impact.

Variable	Pre-UIFSM	Post-UIFSM	Difference	P[1]	Model 1[2]	P	Model 2[3]	P	Model 3[4]	P
	% taking	% taking	Diff. (95% CI)		DID (95% CI)		DID (95% CI)		DID (95% CI)	
Wholemeal (%)										
Intervention	27.0	13.1	-13.9 (-21.6,-6.3)	<0.01	-11.8 (-24.3,0.7)	0.06	-11.8 (-24.0,0.4)	0.06	-12.2 (-24.3,0.0)	0.05
Control	27.3	25.1	-2.1 (-12.0,7.7)	0.67						
Starchy foods (no oil) (%)										
Intervention	75.4	84.9	9.5 (1.6,17.4)	0.02	0.4 (-11.8,12.7)	0.94	0.5 (-11.6,12.7)	0.93	0.6 (-11.6,12.8)	0.92
Control	71.3	80.4	9.0 (-0.3,18.4)	0.06						
Starchy foods (cooked in oil) (%)										
Intervention	31.7	36.9	5.2 (-4.3,14.7)	0.28	4.7 (-9.3,18.6)	0.51	5.0 (-8.6,18.6)	0.47	5.4 (-8.1,19.0)	0.43
Control	31.7	32.3	0.5 (-9.8,10.9)	0.92						
Biscuit (%)										
Intervention	35.6	33.8	-1.8 (-11.6,8.1)	0.73	-3.1 (-17.8,11.5)	0.67	-2.6 (-16.7,11.5)	0.72	-2.7 (-16.8,11.5)	0.71
Control	41.8	43.2	1.4 (-9.5,12.2)	0.80						
Crisps (%)										
Intervention	22.1	11.1	-11.0 (-18.3,-3.7)	<0.01	-17.8 (-30.3,-5.3)	0.01	-17.6 (-30.0,-5.2)	0.01	-18.1 (-30.5,-5.7)	<0.01
Control	24.4	31.2	6.8 (-3.3,17.0)	0.19						
Puddings (%)										
Intervention	44.5	60.5	16.0 (5.9,26.2)	<0.01	14.1 (-0.9,29.0)	0.07	14.5 (-0.2,29.2)	0.05	14.0 (-0.7,28.7)	0.06
Control	45.7	47.6	1.9 (-9.0,12.9)	0.73						
Confectionery (%)										
Intervention	9.6	9.7	0.0 (-6.2,6.3)	0.99	-3.6 (-13.2,6.0)	0.46	-3.7 (-13.1,5.7)	0.44	-3.7 (-13.1,5.6)	0.44
Control	12.5	16.2	3.7 (-3.7,11.0)	0.33						
Sugar-sweetened beverages (%)										
Intervention	14.2	8.0	-6.3 (-13.2,0.6)	0.08	-2.2 (-11.9,7.6)	0.66	-2.3 (-12.0,7.3)	0.64	-2.6 (-12.3,7.1)	0.60
Control	14.5	10.3	-4.1 (-11.0,2.8)	0.25						
Dairy (%)										
Intervention	70.5	66.1	-4.4 (-14.0,5.3)	0.38	-12.8 (-26.7,1.1)	0.07	-12.8 (-26.4,0.8)	0.07	-13.4 (-27.0,0.2)	0.05

Variable	Pre-UIFSM	Post-UIFSM	Difference	P[1]	Model 1[2]	P	Model 2[3]	P	Model 3[4]	P
	% taking	% taking	Diff. (95% CI)		DID (95% CI)		DID (95% CI)		DID (95% CI)	
Control	65.8	74.3	8.4 (-1.5,18.4)	0.10						
**Fruit and vegetables (%)**										
Intervention	95.0	95.4	0.4 (-4.6,5.4)	0.88	1.3 (-7.1,9.6)	0.77	1.6 (-6.5,9.6)	0.70	1.0 (-6.9,8.8)	0.81
Control	91.1	90.2	-0.9 (-7.5,5.8)	0.80						
**High in saturated fat (%)**										
Intervention	87.9	83.0	-4.9 (-12.1,2.2)	0.18	-7.0 (-17.2,3.2)	0.18	-6.9 (-16.7,2.9)	0.17	-7.5 (-17.2,2.2)	0.13
Control	86.5	88.6	2.1 (-5.2,9.4)	0.57						
**High in sodium (%)**										
Intervention	69.8	61.5	-8.3 (-18.2,1.7)	0.10	-10.4 (-24.5,3.8)	0.15	-10.1 (-23.9,3.6)	0.15	-10.8 (-24.4,2.9)	0.12
Control	70.9	73.0	2.1 (-7.9,12.1)	0.68						
**High in sugar (%)**										
Intervention	66.2	71.2	5.0 (-4.5,14.5)	0.30	-0.4 (-14.0,13.3)	0.96	-0.1 (-13.3,13.1)	0.99	-0.4 (-13.7,12.8)	0.95
Control	71.3	76.7	5.4 (-4.4,15.2)	0.28						
**Milk (%)**										
Intervention	8.2	18.5	10.3 (2.6,18.0)	0.01	9.5 (0.4,18.6)	0.04	9.3 (0.5,18.2)	0.04	9.2 (0.3,18.0)	0.04
Control	5.2	6.0	0.8 (-4.0,5.6)	0.74						
**Yoghurt (%)**										
Intervention	39.9	26.3	-13.5 (-22.9,-4.2)	<0.01	-11.2 (-25.1,2.6)	0.11	-11.0 (-24.7,2.6)	0.11	-11.6 (-25.3,2.0)	0.10
Control	34.0	31.7	-2.3 (-12.5,7.9)	0.66						
**Cheese (%)**										
Intervention	50.5	50.3	-0.3 (-10.6,10.1)	0.96	-12.3 (-27.4,2.7)	0.11	-12.4 (-27.3,2.4)	0.10	-12.6 (-27.5,2.2)	0.10
Control	46.1	58.1	12.1 (1.2,22.9)	0.03						
**Meat, fish and eggs (%)**										
Intervention	80.8	80.6	-0.2 (-8.9,8.5)	0.96	-1.0 (-12.8,10.8)	0.86	-0.8 (-12.4,10.8)	0.89	-1.1 (-12.7,10.4)	0.85
Control	84.1	84.9	0.8 (-7.2,8.9)	0.84						
**Baked beans (%)**										
Intervention	14.2	23.4	9.2 (0.9,17.6)	0.03	4.8 (-6.0,15.5)	0.38	4.8 (-5.6,15.3)	0.36	4.8 (-5.7,15.2)	0.37

Variable	Pre-UIFSM	Post-UIFSM	Difference	P[1]	Model 1[2]	P	Model 2[3]	P	Model 3[4]	P
	% taking	% taking	Diff. (95% CI)		DID (95% CI)		DID (95% CI)		DID (95% CI)	
Control	8.8	13.2	4.4 (-2.4,11.2)	0.20						
**Fruit juice (%)**										
Intervention	65.8	51.8	-14.0 (-24.2,-3.8)	0.01	-10.9 (-25.5,3.7)	0.14	-11.1 (-25.5,3.2)	0.13	-12.0 (-26.2,2.2)	0.10
Control	66.9	63.8	-3.1 (-13.6,7.4)	0.56						
**Fruit (%)**										
Intervention	77.2	72.1	-5.1 (-14.5,4.2)	0.28	0.9 (-12.6,14.4)	0.89	1.2 (-12.0,14.5)	0.86	0.2 (-12.8,13.2)	0.98
Control	76.0	69.9	-6.0 (-15.8,3.7)	0.22						
**Vegetables (%)**										
Intervention	71.2	78.9	7.7 (-1.6,17.0)	0.10	2.5 (-11.3,16.4)	0.72	3.1 (-10.2,16.3)	0.65	2.8 (-10.5,16.1)	0.68
Control	64.8	70.0	5.2 (-5.1,15.5)	0.33						
**Water (%)**										
Intervention	53.4	62.8	9.4 (-1.0,19.8)	0.08	5.8 (-9.3,20.9)	0.45	6.1 (-8.4,20.7)	0.41	4.7 (-9.5,18.8)	0.52
Control	53.1	56.7	3.6 (-7.3,14.6)	0.51						

[1] Survey adjusted t-test; [2] Model 1 - Unadjusted linear probability regression; [3] Model 2 - Linear probability regression adjusted for age, sex, ethnicity, household size, region, household income and IMD; [4] Model 3 - Linear probability regression additionally adjusted for total lunchtime intake (grams). Note: UIFSM -Universal Infant Free School Meal; DID – Difference-in-differences SD – standard deviation; Diff. – Difference; CI - confidence interval; Intervention – Infants (4-7 years); Control – Juniors (8-11 years).

Table S4. Characteristics of schoolchildren (n=854) in England and Scotland before and after the UIFSM policy with IPW applied.

Variable		Pre-UIFSM (2010-2014)[1]			Post-UIFSM (2014-2017)		
		Intervention group: Infants (N=281)	Control group: Juniors (N=239)	P value[2]	Intervention group: Infants (N=172)	Control group: Juniors (N=162)	P value[3]
**Age**	Mean (SD)	5.6 (1.0)	9.1 (1.0)	<0.001b	5.7 (1.0)	9.3 (1.0)	<0.00 b
**Sex**	n (%)			0.71a			0.97 a
Male		144 (51.2)	132 (53.0)		84 (51.0)	90 (50.7)	
Female		137 (48.8)	107 (47.0)		88 (49.0)	72 (49.3)	
**Ethnicity**	n (%)			0.96 a			0.81 a
White		229 (81.5)	196 (81.7)		142 (80.7)	135 (81.9)	
BAME		52 (18.5)	43 (18.3)		30 (19.3)	27 (18.1)	
**Household income (£)**	Mean (SD)	32405.8 (20841.8)	32778.8 (22295.7)	0.87 b	32182.7 (20667.9)	32699.4 (20802.0)	0.84b
**Index of multiple deprivation (quintiles)**	n (%)			0.99 a			0.99 a
Least deprived		62 (22.1)	42 (22.2)		40 (21.2)	42 (21.5)	
2		49 (17.4)	41 (15.6)		29 (16.5)	29 (15.9)	
3		60 (21.4)	44 (21.7)		33 (21.1)	27 (23.0)	
4		64 (22.8)	52 (23.2)		34 (25.3)	32 (23.3)	
Most deprived		46 (16.4)	60 (17.4)		36 (15.9)	32 (16.3)	
**Region**	n (%)			0.98 a			0.88 a
England: North		57 (20.3)	62 (19.6)		44 (19.2)	37 (20.6)	
England: Central		44 (15.7)	42 (16.6)		28 (16.4)	25 (16.0)	
England: South		126 (44.8)	83 (45.7)		87 (43.9)	77 (46.5)	
Scotland		54 (19.2)	52 (18.0)		13 (20.6)	23 (17.0)	
**School lunch preference**	n (%)			0.90 a			<0.001 a
School meal		139 (49.5)	121 (48.9)		141 (78.8)	78 (48.5)	
Packed lunch		142 (50.5)	118 (51.1)		31 (21.2)	84 (51.5)	

[1] Threshold is September 2014 for English participants and January 2015 for Scottish participants; [2] Pre-UIFSM intervention vs Pre-UIFSM control; [3] Post-UIFSM intervention vs Post-UIFSM control; a Chi-square test (Adjusted for survey weights); b t-test Adjusted for survey weights) Note: UIFSM -Universal Infant Free School Meal; SD – standard deviation .

Table S5. Mean lunchtime food group consumption, conditional on consumption, in schoolchildren before and after UIFSM implementation and estimates of UIFSM policy impact.

Variable	N	Pre-UIFSM	Post-UIFSM	Difference	P[1]	Model 1[2]	P	Model 2[3]	P	Model 3[4]	P
	Pre / Post	Mean (SD)	Mean (SD)			Coef. (95% CI)		Coef. (95% CI)		Coef. (95% CI)	
Total lunchtime intake (g)											
Intervention	281/172	387.7 (120.3)	408.7 (147.7)	21.0 (-6.5,48.6)	0.14	19.3 (-28.4,67.1)	0.43	20.1 (-27.2,67.5)	0.40	-	-
Control	239/162	428.6 (156.3)	430.3 (180.4)	1.7 (-37.4,40.7)	0.93						
Wholemeal (g)											
Intervention	76/23	39.1 (21.7)	30.8 (18.2)	-8.2 (-17.6,1.2)	0.09	-13.5 (-29.5,2.6)	0.10	-14.0 (-29.4,1.4)	0.08	-13.7 (-28.5,1.1)	0.07
Control	58/40	41.1 (26.4)	46.3 (30.8)	5.3 (-7.8,18.3)	0.43						
Starchy foods (no oil) (g)											
Intervention	212/146	54.2 (28.4)	62.9 (35.2)	8.7 (1.7,15.7)	0.02	6.1 (-5.3,17.4)	0.30	6.1 (-5.2,17.4)	0.29	5.3 (-5.8,16.4)	0.35
Control	175/130	61.3 (33.6)	63.9 (37.3)	2.6 (-6.3,11.5)	0.56						
Starchy foods (cooked in oil) (g)											
Intervention	89/74	39.7 (22.2)	39.0 (21.4)	-0.7 (-7.4,6.1)	0.84	6.2 (-4.7,17.1)	0.27	5.8 (-5.2,16.8)	0.30	5.8 (-5.2,16.8)	0.30
Control	75/50	42.5 (23.4)	35.6 (20.3)	-6.9 (-15.4,1.7)	0.12						
Biscuit (g)											
Intervention	100/60	16.5 (11.1)	15.1 (9.7)	-1.4 (-4.9,2.0)	0.42	-3.3 (-8.1,1.5)	0.18	-3.7 (-8.4,0.9)	0.12	-3.7 (-8.3,0.9)	0.12
Control	97/72	16.4 (10.4)	18.3 (10.7)	1.8 (-1.5,5.1)	0.28						
Crisps (g)											
Intervention	62/18	14.4 (8.0)	17.6 (8.4)	3.1 (-1.5,7.8)	0.19	0.5 (-5.2,6.2)	0.86	1.0 (-4.6,6.5)	0.73	1.0 (-4.6,6.5)	0.73
Control	52/50	14.4 (6.9)	17.0 (8.8)	2.6 (-0.6,5.9)	0.11						
Puddings (g)											
Intervention	125/103	45.4 (30.2)	59.3 (53.6)	13.9 (-3.2,31.0)	0.11	3.5 (-16.4,23.3)	0.73	2.0 (-14.0,18.0)	0.81	1.6 (-14.0,17.3)	0.84
Control	108/77	37.3 (28.9)	47.7 (34.1)	10.4 (0.3,20.5)	0.04						
Confectionery (g)											

Variable	N	Pre-UIFSM	Post-UIFSM	Difference	P[1]	Model 1[2]	P	Model 2[3]	P	Model 3[4]	P
	Pre / Post	Mean (SD)	Mean (SD)			Coef. (95% CI)		Coef. (95% CI)		Coef. (95% CI)	
Intervention	27/16	12.8 (7.0)	12.4 (9.3)	-0.4 (-5.6,4.8)	0.89	3.8 (-4.0,11.5)	0.34	2.6 (-4.7,9.9)	0.49	2.7 (-5.0,10.5)	0.49
Control	34/27	16.9 (13.0)	12.7 (8.5)	-4.1 (-9.9,1.6)	0.16						
Sugar-sweetened beverages (g)											
Intervention	40/10	167.4 (78.6)	110.2 (72.2)	-57.2 (-108.4,-6.0)	0.03	-29.7 (-106.9,47.5)	0.45	-34.2 (-112.0,43.7)	0.39	-47.7 (-116.3,20.8)	0.18
Control	38/16	190.8 (97.9)	163.2 (90.4)	-27.6 (-85.4,30.3)	0.35						
Dairy (g)											
Intervention	198/113	50.8 (48.0)	62.5 (63.7)	11.7 (-4.8,28.2)	0.17	23.1 (3.0,43.2)	0.02	21.6 (2.9,40.3)	0.02	20.4 (1.7,39.0)	0.03
Control	160/121	48.4 (52.7)	37.1 (36.1)	-11.4 (-22.9,0.1)	0.05						
Fruit and vegetables (g)											
Intervention	267/165	61.8 (42.8)	62.1 (45.2)	0.3 (-9.5,10.0)	0.96	4.5 (-9.7,18.8)	0.53	4.2 (-9.9,18.2)	0.56	2.2 (-11.4,15.7)	0.75
Control	216/148	64.4 (45.8)	60.1 (44.3)	-4.2 (-14.6,6.2)	0.42						
High in saturated fat (g)											
Intervention	247/142	38.9 (26.7)	38.2 (24.3)	-0.7 (-6.4,5.0)	0.81	0.5 (-7.6,8.7)	0.90	0.5 (-7.4,8.4)	0.90	0.3 (-7.5,8.2)	0.94
Control	208/142	41.3 (27.0)	40.1 (24.1)	-1.2 (-7.0,4.6)	0.68						
High in sodium (g)											
Intervention	196/108	36.6 (27.4)	26.2 (18.2)	-10.4 (-15.9,-4.9)	<0.01	-8.3 (-16.2,-0.4)	0.04	-9.1 (-16.7,-1.5)	0.02	-9.1 (-16.6,-1.6)	0.02
Control	165/124	34.5 (25.7)	32.4 (22.0)	-2.1 (-7.8,3.6)	0.48						
High in sugar (g)											
Intervention	186/123	35.0 (36.9)	32.2 (22.5)	-2.8 (-9.8,4.1)	0.42	-1.6 (-12.2,9.1)	0.77	-1.9 (-12.5,8.7)	0.73	-2.5 (-13.0,8.0)	0.64
Control	175/123	35.6 (36.7)	34.3 (33.7)	-1.3 (-9.3,6.7)	0.75						
Milk (g)											
Intervention	23/28	106.5 (51.2)	120.6 (59.7)	14.2 (-18.7,47.0)	0.40	74.1 (8.9,139.3)	0.03	57.6 (-5.5,120.6)	0.08	37.4 (-23.4,98.3)	0.23
Control	15/11	135.8 (75.1)	75.9 (50.6)	-60.0 (-116.3,-3.6)	0.05						
Yoghurt (g)											
Intervention	112/47	52.1 (32.5)	47.6 (24.0)	-4.5 (-14.6,5.5)	0.38	5.3 (-11.1,21.7)	0.53	5.1 (-11.6,21.8)	0.55	3.4 (-13.0,19.9)	0.68
Control	82/55	58.4 (40.2)	48.6 (32.6)	-9.8 (-22.8,3.2)	0.14						
Cheese (g)											

Variable	N	Pre-UIFSM	Post-UIFSM	Difference	P[1]	Model 1[2]	P	Model 2[3]	P	Model 3[4]	P
	Pre / Post	Mean (SD)	Mean (SD)			Coef. (95% CI)		Coef. (95% CI)		Coef. (95% CI)	
Intervention	142/87	12.5 (10.0)	12.9 (10.9)	0.3 (-3.1,3.8)	0.85	-1.8 (-6.3,2.6)	0.42	-2.2 (-6.3,1.9)	0.29	-2.4 (-6.4,1.7)	0.26
Control	109/90	10.8 (10.3)	13.0 (9.2)	2.2 (-0.6,4.9)	0.12						
Meat, fish and eggs (g)											
Intervention	227/143	31.7 (21.6)	31.4 (16.7)	-0.3 (-4.4,3.8)	0.88	2.3 (-3.8,8.5)	0.46	2.7 (-3.3,8.7)	0.38	2.4 (-3.5,8.3)	0.43
Control	196/140	33.7 (21.9)	31.1 (18.4)	-2.7 (-7.3,1.9)	0.26						
Baked beans (g)											
Intervention	40/40	34.8 (20.4)	39.4 (25.1)	4.6 (-5.4,14.5)	0.37	-7.2 (-32.3,17.9)	0.57	-6.9 (-29.5,15.8)	0.55	-6.3 (-28.4,15.8)	0.58
Control	24/21	35.0 (25.2)	46.8 (40.4)	11.8 (-11.2,34.8)	0.32						
Fruit juice (g)											
Intervention	185/85	44.4 (61.3)	28.0 (52.5)	-16.3 (-31.6,-1.1)	0.04	-7.8 (-31.6,16.1)	0.52	-9.0 (-33.5,15.4)	0.47	-10.7 (-35.3,13.8)	0.39
Control	161/106	51.8 (68.5)	43.2 (63.6)	-8.6 (-26.9,9.7)	0.36						
Fruit (g)											
Intervention	217/126	41.9 (37.4)	37.1 (38.0)	-4.7 (-13.8,4.3)	0.31	-1.5 (-15.7,12.6)	0.83	-1.5 (-15.6,12.5)	0.83	-0.9 (-14.7,13.0)	0.90
Control	173/117	45.8 (46.0)	42.6 (38.9)	-3.2 (-14.0,7.7)	0.56						
Vegetables (g)											
Intervention	200/139	37.1 (28.6)	41.1 (27.8)	4.1 (-2.7,10.9)	0.24	5.9 (-4.3,16.2)	0.26	6.0 (-4.0,16.0)	0.24	4.8 (-4.9,14.5)	0.33
Control	155/115	36.8 (27.7)	35.0 (28.5)	-1.9 (-9.5,5.8)	0.64						
Water (g)											
Intervention	150/118	125.2 (87.2)	149.5 (113.5)	24.3 (-1.1,49.7)	0.06	16.4 (-36.8,69.7)	0.55	18.7 (-28.8,66.3)	0.44	12.1 (-18.7,43.0)	0.44
Control	121/88	167.1 (114.6)	174.9 (166.0)	7.9 (-39.0,54.7)	0.74						

[1] Survey adjusted t-test; [2] Model 1 - Unadjusted linear probability regression; [3] Model 2 - Linear probability regression adjusted for age, sex, ethnicity, household size, region, household income and IMD; [4] Model 3 - Linear probability regression additionally adjusted for total lunchtime intake (grams). Note: UIFSM - Universal Infant Free School Meal; DID – Difference-in-differences SD – standard deviation; Diff. – Difference; CI - confidence interval; Intervention – Infants (4-7 years); Control – Juniors (8-11 years).

Table S6. Mean lunchtime nutrient intakes in schoolchildren before and after UIFSM implementation and estimates of UIFSM policy impact.

Variable	Pre-UIFSM	Post-UIFSM	Difference	P[1]	Model 1[2]	P	Model 2[3]	P	Model 3[4]	P
	Mean (SD)	Mean (SD)	Diff. (95% CI)		Coef. (95% CI)		Coef. (95% CI)		Coef. (95% CI)	
**Energy (Kcal)**										
Intervention	410.9 (131.0)	411.0 (133.5)	0.1 (-28.7,28.8)	1.00	-24.7 (-63.8,14.5)	0.22	-24.6 (-62.7,13.6)	0.21	-31.9 (-66.4,2.6)	0.07
Control	447.4 (140.6)	472.2 (118.8)	24.7 (-1.8,51.3)	0.07						
**Total fat (g)**										
Intervention	15.7 (7.5)	15.2 (6.9)	-0.5 (-2.0,1.0)	0.52	-2.2 (-4.3,-0.1)	0.04	-2.2 (-4.3,-0.2)	0.03	-2.5 (-4.5,-0.5)	0.01
Control	16.5 (7.3)	18.2 (7.1)	1.7 (0.3,3.2)	0.02						
**Saturated fat (g)**										
Intervention	5.9 (3.2)	5.7 (3.3)	-0.2 (-0.9,0.5)	0.59	-0.6 (-1.5,0.4)	0.23	-0.6 (-1.5,0.3)	0.21	-0.7 (-1.5,0.2)	0.14
Control	6.0 (3.3)	6.4 (2.8)	0.4 (-0.2,1.0)	0.22						
**Carbohydrate (g)**										
Intervention	55.2 (18.3)	56.0 (18.6)	0.7 (-3.2,4.7)	0.71	-0.8 (-6.5,4.8)	0.77	-0.8 (-6.3,4.7)	0.77	-1.9 (-7.0,3.2)	0.46
Control	62.2 (21.1)	63.8 (17.8)	1.6 (-2.5,5.7)	0.44						
**NMES (g)**										
Intervention	13.7 (10.0)	12.7 (10.2)	-1.0 (-3.2,1.2)	0.36	0.1 (-3.1,3.2)	0.97	0.0 (-3.0,3.1)	0.98	-0.3 (-3.2,2.7)	0.84
Control	15.7 (11.2)	14.6 (10.1)	-1.1 (-3.3,1.2)	0.35						
**Protein (g)**										
Intervention	15.7 (5.5)	16.1 (6.0)	0.5 (-0.8,1.7)	0.46	-0.4 (-2.1,1.4)	0.67	-0.4 (-2.1,1.3)	0.67	-0.7 (-2.2,0.9)	0.42
Control	16.4 (6.5)	17.3 (5.1)	0.9 (-0.4,2.1)	0.17						
**Sodium (mg)**										
Intervention	515.7 (241.9)	453.0 (207.1)	-62.7 (-107.1,-18.2)	0.01	-94.7 (-159.1,-30.3)	<0.01	-94.4 (-157.2,-31.5)	<0.01	-103.8 (-163.1,-44.5)	<0.01
Control	534.6 (233.4)	566.6 (212.9)	32.0 (-14.7,78.7)	0.18						
**Fibre (g)**										
Intervention	4.3 (1.9)	4.4 (1.6)	0.1 (-0.2,0.5)	0.52	-0.2 (-0.8,0.3)	0.44	-0.2 (-0.7,0.3)	0.46	-0.3 (-0.8,0.2)	0.26

Variable	Pre-UIFSM	Post-UIFSM	Difference	P[1]	Model 1[2]	P	Model 2[3]	P	Model 3[4]	P
	Mean (SD)	Mean (SD)	Diff. (95% CI)		Coef. (95% CI)		Coef. (95% CI)		Coef. (95% CI)	
Control	4.4 (1.9)	4.8 (2.0)	0.3 (-0.1,0.8)	0.13						
**Calcium (mg)**										
Intervention	209.8 (133.0)	206.5 (131.3)	-3.3 (-32.5,25.8)	0.82	-25.0 (-63.2,13.2)	0.20	-26.2 (-62.7,10.2)	0.16	-30.5 (-66.1,5.1)	0.09
Control	200.9 (119.6)	222.5 (108.1)	21.7 (-3.1,46.4)	0.09						
**Iron (mg)**										
Intervention	2.0 (0.7)	1.9 (0.7)	-0.0 (-0.2,0.1)	0.68	-0.1 (-0.3,0.1)	0.50	-0.1 (-0.3,0.1)	0.52	-0.1 (-0.3,0.1)	0.33
Control	2.2 (0.8)	2.2 (0.7)	0.0 (-0.1,0.2)	0.59						
**Zinc (mg)**										
Intervention	1.7 (0.7)	1.8 (0.9)	0.1 (-0.1,0.3)	0.24	-0.0 (-0.3,0.3)	0.99	-0.0 (-0.3,0.2)	0.97	-0.0 (-0.3,0.2)	0.72
Control	1.8 (0.8)	1.9 (0.7)	0.1 (-0.0,0.3)	0.14						
**Potassium (mg)**										
Intervention	592.6 (228.5)	641.7 (257.3)	49.1 (-0.9,99.1)	0.05	32.3 (-41.0,105.6)	0.39	33.1 (-39.4,105.5)	0.37	20.4 (-45.7,86.4)	0.55
Control	611.7 (262.5)	628.6 (227.7)	16.8 (-36.9,70.5)	0.54						
**Folate (ug)**										
Intervention	45.5 (22.6)	41.7 (20.4)	-3.8 (-8.1,0.4)	0.08	-2.9 (-9.7,4.0)	0.41	-2.8 (-9.5,3.9)	0.41	-3.8 (-10.1,2.4)	0.23
Control	46.0 (27.2)	45.0 (21.9)	-1.0 (-6.3,4.4)	0.73						
**Vitamin C (mg)**										
Intervention	23.4 (20.7)	19.4 (18.8)	-4.0 (-8.2,0.2)	0.06	-3.7 (-9.9,2.5)	0.24	-3.9 (-10.0,2.3)	0.22	-4.6 (-10.5,1.4)	0.13
Control	24.3 (21.5)	24.0 (20.2)	-0.3 (-4.9,4.3)	0.90						
**Vitamin A (ug)**										
Intervention	225.1 (274.4)	193.7 (207.1)	-31.4 (-79.0,16.2)	0.20	-61.4 (-122.6,-0.2)	0.05	-59.7 (-120.0,0.7)	0.05	-63.9 (-123.8,-4.1)	0.04
Control	147.0 (171.6)	177.0 (197.9)	30.0 (-8.6,68.6)	0.13						
**Fat (% of Energy)**										
Intervention	33.2 (8.7)	32.3 (8.5)	-0.8 (-2.6,0.9)	0.35	-2.5 (-4.9,0.0)	0.05	-2.4 (-4.9,-0.0)	0.05	-2.4 (-4.9,0.0)	0.05
Control	32.7 (8.4)	34.3 (7.3)	1.6 (-0.1,3.3)	0.06						

Variable	Pre-UIFSM	Post-UIFSM	Difference	P[1]	Model 1[2]	P	Model 2[3]	P	Model 3[4]	P
	Mean (SD)	Mean (SD)	Diff. (95% CI)		Coef. (95% CI)		Coef. (95% CI)		Coef. (95% CI)	
Saturated Fat (% of Energy)										
Intervention	12.4 (4.8)	11.9 (4.8)	-0.5 (-1.5,0.5)	0.32	-0.8 (-2.2,0.5)	0.21	-0.8 (-2.1,0.5)	0.20	-0.9 (-2.2,0.4)	0.20
Control	11.7 (4.7)	12.0 (3.7)	0.3 (-0.5,1.2)	0.46						
Carbohydrate (% of Energy)										
Intervention	53.8 (10.5)	55.1 (9.2)	1.3 (-0.7,3.3)	0.21	2.8 (-0.1,5.6)	0.06	2.8 (-0.0,5.6)	0.05	2.7 (-0.1,5.5)	0.06
Control	55.6 (10.2)	54.2 (8.2)	-1.5 (-3.4,0.5)	0.15						
NMES (% of Energy)										
Intervention	12.9 (8.7)	11.4 (7.7)	-1.5 (-3.1,0.2)	0.09	0.2 (-2.3,2.6)	0.90	0.2 (-2.2,2.5)	0.90	0.0 (-2.3,2.4)	0.97
Control	13.6 (8.8)	11.9 (7.4)	-1.6 (-3.4,0.1)	0.07						
Protein (% of Energy)										
Intervention	15.7 (6.0)	16.1 (5.1)	0.4 (-0.7,1.4)	0.50	0.1 (-1.3,1.5)	0.85	0.1 (-1.2,1.5)	0.84	0.1 (-1.2,1.5)	0.83
Control	14.8 (4.5)	15.0 (4.2)	0.2 (-0.7,1.1)	0.62						

[1] Survey adjusted t-test; [2] Model 1 - Unadjusted linear probability regression; [3] Model 2 - Linear probability regression adjusted for age sex, ethnicity, household size, region, household income and IMD; [4] Model 3 - Linear probability regression additionally adjusted for total lunchtime intake (grams). Note: UIFSM -Universal Infant Free School Meal; DID – Difference-in-differences SD – standard deviation; Diff. – Difference; CI - confidence interval; Intervention – Infants (4-7 years); Control – Juniors (8-11 years); NMES – non-milk extrinsic sugars.

Table S7. Difference-in-differences estimates for the impact of UIFSM on food-group outcomes using different specifications of IPW and level of adjustment.

Variable	no IPW<br>Unadjusted	P	no IPW<br>Adjusted	P	IPW<br>Unadjusted	P	IPW<br>Adjusted	[illegible]
Wholemeal	-0.14 (-0.27,-0.02)	0.03	-0.12 (-0.24,0.00)	0.05	-0.12 (-0.24,0.01)	0.06	-0.12 (-0.24,0.00)	[illegible]
Starchy foods (no oil)	0.10 (-0.02,0.22)	0.11	0.08 (-0.04,0.21)	0.18	0.00 (-0.12,0.13)	0.94	0.01 (-0.12,0.13)	[illegible]
Crisps	-0.18 (-0.31,-0.05)	0.01	-0.19 (-0.32,-0.07)	<0.01	-0.18 (-0.30,-0.05)	0.01	-0.18 (-0.30,-0.06)	[illegible]
Puddings	0.22 (0.07,0.38)	<0.01	0.20 (0.05,0.35)	0.01	0.14 (-0.01,0.29)	0.07	0.14 (-0.01,0.29)	[illegible]
Dairy	-0.14 (-0.28,0.00)	0.05	-0.15 (-0.29,-0.01)	0.03	-0.13 (-0.27,0.01)	0.07	-0.13 (-0.27,0.00)	[illegible]

Fruit and vegetables	0.03 (-0.05,0.10)	0.51	0.02 (-0.06,0.09)	0.68	0.01 (-0.07,0.10)	0.77	0.01 (-0.07,0.09)	0
High in saturated fat	-0.04 (-0.15,0.07)	0.50	-0.05 (-0.16,0.06)	0.35	-0.07 (-0.17,0.03)	0.18	-0.08 (-0.17,0.02)	0
High in sodium	-0.16 (-0.30,-0.02)	0.02	-0.16 (-0.30,-0.03)	0.02	-0.10 (-0.24,0.04)	0.15	-0.11 (-0.24,0.03)	0
High in sugar	0.07 (-0.07,0.21)	0.32	0.05 (-0.09,0.19)	0.46	-0.00 (-0.14,0.13)	0.96	-0.00 (-0.14,0.13)	0

Note:"no IPW" – Models accounted for survey weighting, does not include an IPW weight; "IPW" - IPW weight produced using multinomial regression with survey weights included as a predictor. Pre-infants as focal. This weight was used in the main analyses; Unadjusted - Linear probability regression not adjusted for covariates; Adjusted - Linear probability regression adjusted for age, sex, ethnicity, household size, region, household income, IMD and total lunch (g).

Table S8. Difference in difference estimates for the impact of UIFSM on nutrient outcomes using different specifications of IPW and level of adjustment.

Variable	no IPW Unadjusted	P	no IPW Adjusted	P	IPW Unadjusted	P	IPW Adjusted
Energy (Kcal)	-16.2 (-58.3,26.0)	0.45	-26.0 (-63.2,11.1)	0.17	-24.7 (-63.8,14.5)	0.22	-31.9 (-66.4,2.6)
Total fat (g)	-2.6 (-4.9,-0.2)	0.03	-2.9 (-5.2,-0.7)	0.01	-2.2 (-4.3,-0.1)	0.04	-2.5 (-4.5,-0.5)
Carbohydrate (g)	2.7 (-3.6,8.9)	0.40	1.3 (-4.2,6.7)	0.65	-0.8 (-6.5,4.8)	0.77	-1.9 (-7.0,3.2)
NMES (g)	3.1 (-0.1,6.3)	0.06	2.8 (-0.2,5.8)	0.07	0.1 (-3.1,3.2)	0.97	-0.3 (-3.2,2.7)
Protein (g)	-0.8 (-2.5,1.0)	0.40	-1.1 (-2.8,0.5)	0.18	-0.4 (-2.1,1.4)	0.67	-0.7 (-2.2,0.9)
Sodium (mg)	-123.0 (-189.4,-56.7)	<0.01	-126.9 (-187.4,-66.4)	<0.01	-94.7 (-159.1,-30.3)	<0.01	-103.8 (-163.1,-44.5)
Potassium (mg)	48.0 (-26.2,122.1)	0.21	27.6 (-41.3,96.5)	0.43	32.3 (-41.0,105.6)	0.39	20.4 (-45.7,86.4)

Note:"no IPW" – Models accounted for survey weighting, does not include an IPW weight;"IPW" - IPW weight produced using multinomial regression with survey weights included as a predictor. Pre-infants as focal. This weight was used in the main analyses. Unadjusted - Linear probability regression not adjusted for covariates Adjusted - Linear probability regression adjusted for age, sex, ethnicity, household size, region, household income, IMD and total lunch (g).

Table S9. Difference-in-differences estimates for the impact of UIFSM on food-group outcomes after excluding unreliable energy reporters.

Variable	Main analysis Unadjusted	P	Main analysis Adjusted	P	Reliable reporters Unadjusted	P	Reliable reporters Adjusted	P
Wholemeal	-0.12 (-0.24,0.01)	0.06	-0.12 (-0.24,0.00)	0.05	-0.12 (-0.25,0.01)	0.06	-0.12 (-0.25,0.00)	0
Starchy foods (no oil)	0.00 (-0.12,0.13)	0.94	0.01 (-0.12,0.13)	0.92	0.01 (-0.11,0.14)	0.83	0.02 (-0.11,0.14)	0
Crisps	-0.18 (-0.30,-0.05)	0.01	-0.18 (-0.30,-0.06)	<0.01	-0.17 (-0.30,-0.04)	0.01	-0.17 (-0.30,-0.04)	0
Puddings	0.14 (-0.01,0.29)	0.07	0.14 (-0.01,0.29)	0.06	0.12 (-0.04,0.27)	0.13	0.12 (-0.03,0.27)	0

Dairy	-0.13 (-0.27,0.01)	0.07	-0.13 (-0.27,0.00)	0.05	-0.15 (-0.30,-0.01)	0.04	-0.16 (-0.30,-0.02)	0[illegible]
Fruit and vegetables	0.01 (-0.07,0.10)	0.77	0.01 (-0.07,0.09)	0.81	0.01 (-0.08,0.09)	0.84	0.01 (-0.07,0.09)	0[illegible]
High in saturated fat	-0.07 (-0.17,0.03)	0.18	-0.08 (-0.17,0.02)	0.13	-0.05 (-0.15,0.05)	0.30	-0.05 (-0.15,0.04)	0[illegible]
High in sodium	-0.10 (-0.24,0.04)	0.15	-0.11 (-0.24,0.03)	0.12	-0.09 (-0.24,0.05)	0.20	-0.09 (-0.23,0.05)	0[illegible]
High in sugar	-0.00 (-0.14,0.13)	0.96	-0.00 (-0.14,0.13)	0.95	-0.02 (-0.16,0.12)	0.83	-0.01 (-0.15,0.12)	0[illegible]

Note: Reliable reporters = participants whose reported energy was estimated to be under (n=42) and over(n=2) their energy requirement, using the Goldberg method adjusted for children, were excluded from this group, leaving only reliable reporters (n=810); Unadjusted - Linear regression not adjusted for covariates; Adjusted - Linear regression adjusted for sage, ex, ethnicity, household size, region, household income, IMD and total lunch (g).

Table S10. Difference in difference estimates for the impact of UIFSM on nutrient outcomes after excluding unreliable energy reporters.

Variable	Main analysis Unadjusted	P	Main analysis Adjusted	P	Reliable reporters Unadjusted	P	Reliable reporters Adjusted
Energy (Kcal)	-24.7 (-63.8,14.5)	0.22	-31.9 (-66.4,2.6)	0.07	-26.3 (-65.8,13.2)	0.19	-32.5 (-67.5,2.5)
Total fat (g)	-2.2 (-4.3,-0.1)	0.04	-2.5 (-4.5,-0.5)	0.01	-2.1 (-4.3,0.0)	0.05	-2.3 (-4.3,-0.3)
Carbohydrate (g)	-0.8 (-6.5,4.8)	0.77	-1.9 (-7.0,3.2)	0.46	-1.5 (-7.2,4.2)	0.62	-2.4 (-7.6,2.7)
NMES (g)	0.1 (-3.1,3.2)	0.97	-0.3 (-3.2,2.7)	0.84	-0.0 (-3.3,3.2)	0.98	-0.4 (-3.4,2.7)
Protein (g)	-0.4 (-2.1,1.4)	0.67	-0.7 (-2.2,0.9)	0.42	-0.4 (-2.2,1.4)	0.63	-0.7 (-2.3,1.0)
Sodium (mg)	-94.7 (-159.1,-30.3)	<0.01	-103.8 (-163.1,-44.5)	<0.01	-87.8 (-153.7,-21.9)	0.01	-94.2 (-155.0,-33.4)
Potassium (mg)	32.3 (-41.0,105.6)	0.39	20.4 (-45.7,86.4)	0.55	24.3 (-50.4,98.9)	0.52	14.2 (-53.5,81.8)

Note: Reliable reporters = participants whose reported energy was under (n=42) and over (n=2) their estimated energy requirement were excluded from this group, leaving only reliable reporters (n=810); Unadjusted - Linear regression not adjusted for covariates; Adjusted - Linear regression adjusted for sage, ex, ethnicity, household size, region, household income, IMD and total lunch (g).

Table S11. Mean daily nutrient intakes in schoolchildren, on a school day, and estimates of UIFSM policy impact.

Variable	Pre-UIFSM	Post-UIFSM	Difference	P[1]	Crude DID[2]	P	Adjusted DID[3]	P
	Mean (SD)	Mean (SD)	Diff. (95% CI)		Coef. (95% CI)		Coef. (95% CI)	
**Energy (Kcal)**								
Intervention	1407.5 (325.9)	1408.4 (351.6)	0.9 (-80.3,82.1)	0.98	7.4 (-102.3,117.0)	0.90	2.1 (-101.2,105.5)	0.97
Control	1580.8 (361.5)	1574.3 (332.1)	-6.5 (-80.3,67.3)	0.86				

Variable	Pre-UIFSM	Post-UIFSM	Difference	P[1]	Crude DID[2]	P	Adjusted DID[3]	P
	Mean (SD)	Mean (SD)	Diff. (95% CI)		Coef. (95% CI)		Coef. (95% CI)	
**Total fat (g)**								
Intervention	51.7 (16.6)	51.3 (17.6)	-0.4 (-4.4,3.7)	0.86	-1.9 (-7.3,3.5)	0.48	-2.2 (-7.3,3.0)	0.41
Control	56.7 (18.0)	58.3 (15.6)	1.6 (-2.0,5.1)	0.39				
**Saturated fat (g)**								
Intervention	21.0 (7.8)	20.7 (8.4)	-0.3 (-2.2,1.5)	0.73	-0.5 (-2.9,2.0)	0.71	-0.5 (-2.9,1.8)	0.65
Control	22.1 (8.2)	22.2 (7.5)	0.1 (-1.5,1.8)	0.86				
**Carbohydrate (g)**								
Intervention	194.8 (46.3)	196.6 (48.8)	1.7 (-8.9,12.4)	0.75	6.1 (-9.3,21.5)	0.44	5.5 (-9.2,20.1)	0.46
Control	223.0 (51.6)	218.7 (51.3)	-4.4 (-15.5,6.8)	0.44				
**NMES (g)**								
Intervention	49.8 (26.6)	47.1 (23.4)	-2.7 (-7.6,2.2)	0.28	2.3 (-5.5,10.0)	0.57	2.2 (-5.4,9.7)	0.57
Control	60.8 (29.6)	55.8 (25.9)	-4.9 (-11.0,1.1)	0.11				
**Protein (g)**								
Intervention	52.8 (13.9)	52.4 (15.4)	-0.5 (-4.1,3.2)	0.81	0.4 (-4.4,5.2)	0.86	0.3 (-4.3,4.8)	0.91
Control	58.6 (15.1)	57.7 (13.6)	-0.9 (-4.0,2.3)	0.58				
**Sodium (mg)**								
Intervention	1532.3 (467.0)	1414.0 (431.6)	-118.3 (-209.6,-27.0)	0.01	-76.3 (-214.0,61.4)	0.28	-83.1 (-215.1,49.0)	0.22
Control	1720.9 (527.0)	1678.8 (454.1)	-42.1 (-145.3,61.1)	0.42				
**Fibre (g)**								
Intervention	14.2 (4.2)	15.0 (5.4)	0.8 (-0.4,2.0)	0.18	0.6 (-1.0,2.2)	0.49	0.5 (-1.0,2.1)	0.48
Control	15.3 (4.8)	15.6 (5.0)	0.2 (-0.8,1.3)	0.66				
**Calcium (mg)**								
Intervention	810.5 (317.2)	795.2 (325.4)	-15.2 (-83.6,53.1)	0.66	-21.4 (-115.4,72.6)	0.66	-25.5 (-115.0,63.9)	0.58
Control	796.9 (310.5)	803.1 (278.3)	6.2 (-58.4,70.7)	0.85				
**Iron (mg)**								

Variable	Pre-UIFSM	Post-UIFSM	Difference	P[1]	Crude DID[2]	P	Adjusted DID[3]	P
	Mean (SD)	Mean (SD)	Diff. (95% CI)		Coef. (95% CI)		Coef. (95% CI)	
Intervention	7.9 (2.4)	7.9 (2.5)	0.0 (-0.5,0.6)	0.91	0.5 (-0.3,1.4)	0.22	0.5 (-0.3,1.3)	0.21
Control	9.5 (3.1)	9.0 (2.8)	-0.5 (-1.1,0.1)	0.13				
Zinc (mg)								
Intervention	6.1 (2.0)	6.1 (2.2)	-0.0 (-0.6,0.5)	0.98	-0.1 (-0.8,0.6)	0.84	-0.1 (-0.8,0.5)	0.76
Control	6.5 (2.0)	6.6 (2.0)	0.1 (-0.4,0.5)	0.77				
Potassium (mg)								
Intervention	2133.2 (540.5)	2153.6 (610.8)	20.4 (-124.2,165.0)	0.78	84.6 (-111.2,280.3)	0.40	77.9 (-107.5,263.3)	0.41
Control	2259.1 (625.0)	2194.9 (584.9)	-64.2 (-196.3,67.9)	0.34				
Folate (ug)								
Intervention	180.8 (61.8)	173.3 (54.1)	-7.5 (-19.2,4.2)	0.21	5.7 (-14.4,25.7)	0.58	5.2 (-14.0,24.4)	0.60
Control	205.5 (83.0)	192.3 (68.2)	-13.2 (-29.5,3.1)	0.11				
Vitamin C (mg)								
Intervention	84.0 (46.9)	77.9 (40.6)	-6.1 (-14.9,2.7)	0.18	-5.1 (-18.6,8.4)	0.46	-5.2 (-18.5,8.2)	0.45
Control	85.5 (48.7)	84.4 (47.7)	-1.0 (-11.2,9.2)	0.84				
Vitamin A (ug)								
Intervention	663.7 (434.6)	576.3 (403.1)	-87.4 (-169.8,-5.0)	0.04	-94.8 (-215.7,26.2)	0.12	-90.8 (-209.2,27.5)	0.13
Control	551.3 (438.1)	558.7 (419.9)	7.4 (-81.3,96.0)	0.87				
Fat (% of Energy)								
Intervention	32.7 (5.3)	32.4 (5.3)	-0.3 (-1.4,0.8)	0.60	-1.5 (-3.0,0.0)	0.05	-1.5 (-3.0,-0.0)	0.05
Control	32.0 (5.0)	33.2 (4.7)	1.2 (0.2,2.3)	0.03				
Saturated Fat (% of Energy)								
Intervention	13.2 (3.0)	12.9 (3.0)	-0.3 (-0.9,0.3)	0.29	-0.5 (-1.3,0.4)	0.26	-0.5 (-1.3,0.4)	0.26
Control	12.4 (2.9)	12.6 (2.8)	0.2 (-0.4,0.8)	0.58				
Carbohydrate (% of Energy)								
Intervention	55.6 (6.1)	56.2 (6.6)	0.6 (-0.8,1.9)	0.42	1.6 (-0.2,3.5)	0.08	1.6 (-0.2,3.4)	0.08

Variable	Pre-UIFSM	Post-UIFSM	Difference	P[1]	Crude DID[2]	P	Adjusted DID[3]	P
	Mean (SD)	Mean (SD)	Diff. (95% CI)		Coef. (95% CI)		Coef. (95% CI)	
Control	56.6 (5.1)	55.6 (5.7)	-1.1 (-2.3,0.2)	0.09				
NMES (% of Energy)								
Intervention	13.9 (6.1)	13.2 (5.8)	-0.7 (-1.9,0.6)	0.29	0.4 (-1.3,2.1)	0.66	0.4 (-1.3,2.1)	0.63
Control	15.0 (5.6)	14.0 (5.4)	-1.1 (-2.3,0.1)	0.09				
Protein (% of Energy)								
Intervention	15.2 (2.7)	14.9 (3.0)	-0.2 (-0.8,0.4)	0.50	-0.0 (-0.9,0.8)	0.95	-0.0 (-0.9,0.8)	0.96
Control	15.0 (2.8)	14.8 (2.5)	-0.2 (-0.8,0.4)	0.57				

[1] Survey adjusted t-test; [2] Unadjusted linear regression; [3] Linear regression adjusted for age, sex, ethnicity, household size, region, household income and IMD; SD - standard deviation; CI - confidence interval; NMES – non-milk extrinsic sugars

Table S12. Prevalence of food-group consumption in schoolchildren and estimates of UIFSM policy impact stratified by income.

Variable	Low-income Pre-UIFSM	Difference[1]	Mid-income pre-UIFSM	Difference[1]	High-income Pre-UIFSM	Difference[1]	Low-income DID[2]	P	Mid-income DID[2]	P	High-Income DID[2]	P
	% Taking	Diff. (95% CI)	% Taking	Diff. (95% CI)[1]	% Taking	Diff. (95% CI)	Coef. (95% CI)		Coef. (95% CI)		Coef. (95% CI)	
**Wholemeal**												
Intervention	23.2	-21.3* (-0.3,-11.4)	27.8[a]	-10.0 (-24.8,4.9)	29.4[b]	-11.3 (-24.4,1.9)	-25.7 (-40.5,-10.8)	<0.01	-11.9 (-33.8,10.1)	0.29	2.0 (-20.7,24.6)	0.87
Control	9.3	4.0 (-7.7,15.6)	29.4[a]	4.2 (-12.9,21.3)	40.6[b]	-15.1 (-34.0,3.7)						
**Starchy foods (no oil)**												
Intervention	72.0	16.3* (0.0,29.4)	73.2	9.5 (-5.2,24.2)	80.4	4.0 (-8.8,16.8)	-1.6 (-19.9,16.6)	0.86	11.4 (-10.9,33.7)	0.32	-6.2 (-28.6,16.3)	0.59
Control	69.6	18.3* (4.3,32.3)	75.6	-1.4 (-17.6,14.9)	69.6	11.2 (-5.7,28.2)						
**Starchy foods (cooked in oil)**												
Intervention	28.0	10.5 (-6.4,27.3)	30.9	6.8 (-10.2,23.7)	35.3	-0.8 (-16.0,14.5)	31.7 (9.6,53.8)	0.01	0.9 (-22.1,23.9)	0.94	-14.7 (-37.6,8.3)	0.21
Control	37.1	-19.8* (-35.1,-4.6)	26.8	7.0 (-10.1,24.1)	30.9	11.8 (-7.5,31.0)						
**Biscuit**												
Intervention	36.6	-6.7 (-22.8,9.3)	38.1	-5.4 (-23.9,13.0)	32.4	6.3 (-9.6,22.2)	-17.3 (-42.1,7.4)	0.17	-4.1 (-29.2,20.9)	0.75	10.6 (-12.5,33.6)	0.37
Control	37.1	9.6 (-8.9,28.1)	45.3	1.0 (-17.2,19.2)	43.2	-6.2 (-25.5,13.2)						
**Crisps**												
Intervention	24.4	-10.3 (-24.1,3.5)	26.8	-13.5 (-27.6,0.6)	15.7	-9.7* (-18.9,-0.5)	-32.8 (-54.9,-10.6)	<0.01	-17.0 (-38.0,3.9)	0.11	-4.1 (-23.8,15.6)	0.68
Control	17.6	22.4* (5.4,39.5)	28.1	4.9 (-11.8,21.6)	27.3	-5.1 (-23.4,13.2)						

Variable	Low-income Pre-UIFSM	Difference[1]	Mid-income pre-UIFSM	Difference[1]	High-income Pre-UIFSM	Difference[1]	Low-income DID[2]	P	Mid-income DID[2]	P	High-Income DID[2]	P
	% Taking	Diff. (95% CI)	% Taking	Diff. (95% CI)[1]	% Taking	Diff. (95% CI)	Coef. (95% CI)		Coef. (95% CI)		Coef. (95% CI)	
Puddings												
Intervention	46.3	10.9 (-7.1,28.9)	40.2	23.7* (5.6,41.7)	47.1	12.8 (-3.7,29.3)	10.1 (-13.1,33.3)	0.39	25.1 (0.3,49.8)	0.05	5.6 (-19.5,30.7)	0.66
Control	44.4	5.4 (-13.1,24.0)	39.9	0.7 (-17.2,18.5)	51.0	2.4 (-17.7,22.5)						
Confectionery												
Intervention	6.1	4.8 (-5.7,15.3)	14.4	-3.1 (-15.7,9.4)	7.8	-1.1 (-9.4,7.3)	6.5 (-9.4,22.5)	0.42	-11.9 (-30.9,7.2)	0.22	-2.3 (-14.8,10.1)	0.71
Control	13.4	0.6 (-11.4,12.7)	15.9	8.5 (-5.9,23.0)	9.3	-0.2 (-10.4,10.0)						
Sugar-sweetened beverages												
Intervention	13.4	-6.1 (-16.3,4.2)	20.6	-5.7 (-21.4,10.1)	8.8	-7.8* (-13.7,-1.9)	-6.4 (-22.3,9.5)	0.43	1.5 (-16.6,19.6)	0.87	0.8 (-11.4,12.9)	0.90
Control	13.9	0.3 (-12.7,13.4)	16.6	-5.3 (-17.6,6.9)	13.3	-7.1 (-18.3,4.1)						
Dairy												
Intervention	70.7	-4.7 (-21.6,12.2)	72.2	-11.5 (-29.6,6.6)	68.6	3.4 (-11.5,18.2)	-21.6 (-44.1,0.8)	0.06	-12.2 (-35.8,11.3)	0.31	-4.1 (-26.2,18.0)	0.71
Control	66.9	15.2 (0.1,30.2)	62.4	3.9 (-13.9,21.6)	67.5	9.2 (-8.3,26.6)						
Fruit and vegetables												
Intervention	96.3	0.3 (-5.3,6.0)	94.8	-4.9 (-16.9,7.2)	94.1	5.9* (1.3,10.5)	-2.7 (-17.9,12.5)	0.73	-2.7 (-18.1,12.8)	0.73	7.5 (-2.0,16.9)	0.12
Control	86.3	3.7 (-10.8,18.3)	89.4	-2.7 (-14.0,8.7)	96.4	-2.2 (-11.3,6.9)						
High in saturated fat												

Variable	Low-income Pre-UIFSM	Difference[1]	Mid-income pre-UIFSM	Difference[1]	High-income Pre-UIFSM	Difference[1]	Low-income DID[2]	P	Mid-income DID[2]	P	High-Income DID[2]	P
	% Taking	Diff. (95% CI)	% Taking	Diff. (95% CI)[1]	% Taking	Diff. (95% CI)	Coef. (95% CI)		Coef. (95% CI)		Coef. (95% CI)	
Intervention	92.7	-11.3 (-24.4,1.8)	82.5	5.3 (-5.8,16.5)	89.2	-10.0 (-22.6,2.6)	-26.5 (-42.6,-10.5)	<0.01	6.6 (-8.2,21.3)	0.38	-2.4 (-19.7,14.9)	0.79
Control	76.0	16.4* (4.4,28.3)	91.8	-0.4 (-12.2,11.3)	91.3	-8.7 (-22.2,4.8)						
High in sodium												
Intervention	70.7	-10.7 (-28.0,6.7)	72.2	-8.4 (-26.3,9.5)	66.7	-6.3 (-22.5,9.8)	-24.7 (-48.1,-1.3)	0.04	-2.3 (-23.3,18.7)	0.83	-0.4 (-24.3,23.4)	0.97
Control	55.3	14.3 (-4.2,32.8)	83.9[a]	-1.4 (-16.0,13.3)	74.3	-8.8 (-26.9,9.3)						
High in sugar												
Intervention	74.4	-7.6 (-24.5,9.3)	69.1	1.1 (-15.9,18.2)	56.9	19.4* (4.5,34.3)	-14.4 (-35.5,6.8)	0.18	-0.3 (-22.1,21.5)	0.98	10.8 (-10.7,32.3)	0.33
Control	70.6	9.3 (-5.7,24.3)	74.4	2.1 (-13.5,17.7)	69.6	4.8 (-14.2,23.9)						
Milk												
Intervention	3.7	17.4* (0.0,30.0)	6.2	5.1 (-7.3,17.4)	13.7	10.2 (-4.2,24.6)	18.3 (3.7,33.0)	0.01	-2.6 (-17.6,12.4)	0.73	12.9 (-2.2,27.9)	0.09
Control	8.9	-3.7 (-12.3,4.8)	2.5	7.6 (-1.1,16.3)	4.1	-1.8 (-8.5,4.8)						
Yoghurt												
Intervention	37.8	-1.9 (-18.8,14.9)	45.4	-21.4* (-39.1,-3.6)	36.3	-16.1* (-29.7,-2.4)	-15.7 (-39.7,8.4)	0.20	-10.1 (-32.5,12.4)	0.38	-5.3 (-27.8,17.2)	0.64
Control	34.1	12.5 (-5.7,30.7)	35.7	-7.6 (-24.3,9.1)	32.7	-9.0 (-26.8,8.8)						
Cheese												
Intervention	57.3	-4.6 (-22.5,13.2)	45.4	-4.2 (-23.1,14.6)	50.0	7.9 (-8.4,24.1)	-16.2 (-41.5,9.0)	0.21	-11.5 (-37.0,14.0)	0.38	-8.0 (-33.0,17.1)	0.53

Variable	Low-income Pre-UIFSM	Difference[1]	Mid-income pre-UIFSM	Difference[1]	High-income Pre-UIFSM	Difference[1]	Low-income DID[2]	P	Mid-income DID[2]	P	High-Income DID[2]	P
	% Taking	Diff. (95% CI)	% Taking	Diff. (95% CI)[1]	% Taking	Diff. (95% CI)	Coef. (95% CI)		Coef. (95% CI)		Coef. (95% CI)	
Control	45.1	12.4 (-5.8,30.5)	41.5	9.6 (-8.6,27.8)	50.2	16.0 (-3.2,35.3)						
**Meat, fish and eggs**												
Intervention	81.7	-2.6 (-17.5,12.4)	81.4	0.3 (-16.0,16.5)	79.4	1.3 (-12.2,14.7)	-3.4 (-23.5,16.6)	0.74	-0.4 (-21.3,20.5)	0.97	-2.6 (-20.4,15.2)	0.78
Control	81.7	-0.6 (-16.9,15.6)	81.9	1.1 (-13.0,15.2)	87.7	2.4 (-9.5,14.2)						
**Baked beans**												
Intervention	12.2	4.0 (-8.3,16.4)	12.4	19.4* (2.8,36.0)	17.6	3.4 (-9.3,16.1)	10.0 (-5.6,25.6)	0.21	14.2 (-3.2,31.5)	0.11	-9.7 (-27.1,7.7)	0.28
Control	12.6	-5.5 (-15.1,4.1)	8.3	3.3 (-7.6,14.2)	5.9	13.9* (0.3,27.4)						
**Fruit juice**												
Intervention	61.0	-5.7 (-23.6,12.1)	73.2	-18.1 (-36.2,0.0)	62.7	-17.6* (-33.8,-1.3)	-21.4 (-45.2,2.4)	0.08	-16.0 (-40.2,8.2)	0.20	2.4 (-21.7,26.6)	0.84
Control	61.7	13.6 (-2.9,30.1)	69.8	-3.0 (-20.2,14.2)	69.0	-17.8 (-37.3,1.7)						
**Fruit**												
Intervention	80.5	-2.5 (-16.5,11.5)	79.4	-23.1* (-41.4,-4.9)	72.5	11.2 (-2.5,24.8)	-8.6 (-30.8,13.6)	0.45	-17.7 (-41.5,6.0)	0.14	23.2 (3.4,43.0)	0.02
Control	67.3	4.7 (-13.2,22.5)	72.3	-3.0 (-19.5,13.6)	86.0	-17.0* (-33.2,-0.7)						
**Vegetables**												
Intervention	69.5	11.6 (-3.2,26.4)	58.8	11.7 (-7.4,30.7)	84.3[b]	1.6 (-9.4,12.7)	8.3 (-14.3,30.8)	0.47	4.3 (-20.2,28.8)	0.73	-7.1 (-27.3,13.1)	0.49
Control	62.4	2.7 (-15.6,21.0)	57.1	7.6 (-10.1,25.3)	72.4	7.0 (-10.5,24.5)						

Variable	Low-income Pre-UIFSM	Difference[1]	Mid-income pre-UIFSM	Difference[1]	High-income Pre-UIFSM	Difference[1]	Low-income DID[2]	P	Mid-income DID[2]	P	High-Income DID[2]	P
	% Taking	Diff. (95% CI)	% Taking	Diff. (95% CI)[1]	% Taking	Diff. (95% CI)	Coef. (95% CI)		Coef. (95% CI)		Coef. (95% CI)	
Water												
Intervention	53.7	11.2 (-6.2,28.6)	46.4	8.1 (-11.3,27.4)	59.8	10.1 (-6.1,26.2)	22.1 (-1.3,45.5)	0.06	-10.9 (-35.8,13.9)	0.39	-0.9 (-24.2,22.3)	0.94
Control	51.3	-17.7* (-35.2,-0.2)	39.0	19.5* (1.8,37.3)	65.0[b]	8.4 (-10.2,26.9)						

[1] Survey adjusted t-test; [2] Linear probability regression adjusted for age sex, ethnicity, household size, region, household income, IMD and total lunchtime intake (g). * P<0.05; Note: UIFSM -Universal Infant Free School Meal; DID – Difference-in-differences SD – standard deviation; Diff. – Difference; CI - confidence interval; Intervention – Infants (4-7 years); Control – Juniors (8-11 years) SD - standard deviation; CI - confidence interval.

Table S13. Mean lunchtime nutrient intakes in schoolchildren and estimates of UIFSM policy impact stratified by income.

Variable	Low-income Pre-UIFSM	Difference	Mid-income Pre-UIFSM	Difference	High-income Pre-UIFSM	Difference	Low-income DiD[2]	P	Mid-income DiD[2]	P	High-Income DiD[2]	P
	Mean (SD)	Diff. (95% CI)[1]	Mean (SD)	Diff. (95% CI)[1]	Mean (SD)	Diff. (95% CI)	Coef. (95% CI)		Coef. (95% CI)		Coef. (95% CI)	
**Energy (Kcal)**												
Intervention	429.7 (152.9)	-19.4 (-69.2,30.5)	412.2 (130.1)	4.0 (-50.1,58.1)	394.7 (110.4)	11.4 (-32.7,55.5)	-122.7 (-181.7,-63.6)	<0.01	12.5 (-39.1,64.2)	0.63	-0.1 (-58.1,57.8)	1.00
Control	407.1 (140.1)	85.1* (36.1,134.0)	460.8 (132.7)	-8.7 (-50.2,32.8)	471.1 (140.8)	6.5 (-39.6,52.6)						
**Total fat (g)**												
Intervention	17.4 (9.1)	-2.4 (-5.2,0.3)	15.1 (6.7)	0.5 (-2.3,3.3)	14.9 (6.7)	0.1 (-2.3,2.4)	-7.1 (-10.7,-3.4)	<0.01	-0.1 (-3.3,3.1)	0.96	-0.8 (-3.8,2.2)	0.60
Control	15.2 (6.4)	4.3* (1.3,7.3)	17.0 (7.5)	0.6 (-1.9,3.0)	17.2 (7.7)	0.7 (-1.6,3.0)						
**Saturated fat (g)**												
Intervention	6.5 (3.9)	-0.8 (-2.0,0.4)	5.6 (2.9)	0.1 (-1.1,1.4)	5.7 (2.9)	-0.0 (-1.2,1.2)	-2.7 (-4.2,-1.1)	<0.01	0.3 (-1.2,1.8)	0.71	0.2 (-1.3,1.7)	0.78

Variable	Low-income Pre-UIFSM	Difference	Mid-income Pre-UIFSM	Difference	High-income Pre-UIFSM	Difference	Low-income DiD[2]	P	Mid-income DiD[2]	P	High-Income DiD[2]	P
	Mean (SD)	Diff. (95% CI)[1]	Mean (SD)	Diff. (95% CI)[1]	Mean (SD)	Diff. (95% CI)	Coef. (95% CI)		Coef. (95% CI)		Coef. (95% CI)	
Control	5.3 (3.1)	1.6* (0.5,2.8)	6.5 (3.6)	-0.1 (-1.2,1.0)	6.3 (3.4)	-0.3 (-1.3,0.7)						
**Carbohydrate (g)**												
Intervention	55.9 (19.8)	1.0 (-6.0,8.1)	57.3 (19.0)	-0.5 (-7.7,6.7)	52.7 (16.2)	1.5 (-4.5,7.4)	-11.7 (-20.2,-3.1)	0.01	2.4 (-5.0,9.8)	0.53	1.8 (-7.3,10.8)	0.70
Control	56.4 (22.4)	9.9* (3.1,16.7)	64.1 (19.1)	-3.0 (-9.1,3.1)	65.8 (20.5)	-0.9 (-8.5,6.7)						
**NMES (g)**												
Intervention	13.4 (9.7)	0.4 (-3.9,4.7)	15.5 (10.6)	-2.2 (-6.2,1.7)	12.2 (9.5)	-1.2 (-4.0,1.7)	-0.6 (-6.1,5.0)	0.84	0.2 (-4.1,4.5)	0.93	-0.3 (-5.1,4.5)	0.90
Control	15.2 (12.7)	0.6 (-3.5,4.8)	16.8 (11.0)	-2.8 (-6.1,0.5)	15.3 (10.0)	-0.9 (-5.2,3.3)						
**Protein (g)**												
Intervention	15.9 (6.1)	-0.3 (-2.3,1.7)	15.3 (5.2)	0.3 (-1.7,2.4)	15.8 (5.1)	1.4 (-1.1,3.8)	-3.9 (-6.3,-1.5)	<0.01	1.1 (-1.4,3.6)	0.39	0.1 (-3.0,3.2)	0.96
Control	14.8 (6.2)	2.5* (0.5,4.5)	16.9 (6.2)	-0.7 (-2.7,1.3)	17.4b (6.7)	1.0 (-1.1,3.1)						
**Sodium (mg)**												
Intervention	567.6 (302.5)	-141.1* (-229.4,-52.8)	509.2 (234.4)	-38.1 (-120.2,43.9)	480.2 (181.9)	-22.4 (-78.9,34.0)	-331.0 (-434.8,-227.1)	<0.01	17.0 (-86.4,120.5)	0.75	-7.4 (-97.6,82.8)	0.87
Control	442.2 (226.0)	161.0* (85.1,237.0)	603.0[a] (230.3)	-47.0 (-126.4,32.3)	561.0 (219.6)	-12.3 (-93.5,68.9)						
**Fibre (g)**												
Intervention	4.2 (2.0)	-0.0 (-0.6,0.6)	4.2 (1.9)	0.3 (-0.4,0.9)	4.5 (1.7)	0.1 (-0.4,0.6)	-0.6 (-1.4,0.2)	0.13	-0.1 (-1.1,0.8)	0.75	-0.3 (-1.1,0.5)	0.47

Variable	Low-income Pre-UIFSM	Difference	Mid-income Pre-UIFSM	Difference	High-income Pre-UIFSM	Difference	Low-income DiD[2]	P	Mid-income DiD[2]	P	High-Income DiD[2]	P
	Mean (SD)	Diff. (95% CI)[1]	Mean (SD)	Diff. (95% CI)[1]	Mean (SD)	Diff. (95% CI)	Coef. (95% CI)		Coef. (95% CI)		Coef. (95% CI)	
Control	3.6 (1.5)	0.3 (-0.2,0.8)	4.5[a] (2.2)	0.4 (-0.5,1.2)	5.0[b] (1.7)	0.2 (-0.4,0.9)						
**Calcium (mg)**												
Intervention	215.7 (148.1)	-6.7 (-53.4,40.1)	201.8 (129.9)	0.1 (-55.7,56.0)	212.7 (123.8)	-3.7 (-51.3,44.0)	-99.7 (-157.2,-42.2)	<0.01	23.9 (-38.3,86.1)	0.45	-15.3 (-75.9,45.3)	0.62
Control	178.4 (120.8)	72.5* (32.7,112.2)	219.9 (128.9)	-21.7 (-61.5,18.1)	205.5 (109.4)	20.4 (-25.3,66.1)						
**Iron (mg)**												
Intervention	1.9 (0.7)	-0.2 (-0.4,0.1)	1.9 (0.7)	0.0 (-0.2,0.3)	2.1[b] (0.7)	0.0 (-0.2,0.3)	-0.6 (-0.9,-0.3)	<0.01	0.2 (-0.1,0.5)	0.31	0.0 (-0.4,0.4)	0.92
Control	1.9 (0.7)	0.4* (0.1,0.6)	2.2 (0.8)	-0.1 (-0.4,0.2)	2.4[b] (0.9)	-0.0 (-0.3,0.3)						
**Zinc (mg)**												
Intervention	1.8 (0.7)	-0.1 (-0.3,0.2)	1.6 (0.7)	0.2 (-0.2,0.5)	1.8 (0.7)	0.3 (-0.1,0.7)	-0.5 (-0.8,-0.2)	<0.01	0.2 (-0.2,0.6)	0.34	0.1 (-0.3,0.6)	0.58
Control	1.5 (0.7)	0.3* (0.1,0.5)	1.9[a] (0.7)	-0.0 (-0.3,0.2)	1.9[b] (0.9)	0.1 (-0.2,0.4)						
**Potassium (mg)**												
Intervention	584.1 (236.3)	78.6 (-7.3,164.5)	597.2 (214.0)	39.6 (-48.3,127.6)	595.1 (237.5)	32.8 (-51.9,117.5)	37.5 (-66.4,141.5)	0.48	37.3 (-79.2,153.8)	0.53	-43.4 (-153.8,67.0)	0.44
Control	576.9 (259.1)	-8.3 (-83.7,67.2)	621.3 (276.2)	-5.5 (-105.8,94.8)	633.7b (254.8)	56.7 (-34.3,147.6)						
**Folate (ug)**												
Intervention	45.0 (22.4)	-5.3 (-13.0,2.5)	42.9 (21.2)	-0.1 (-7.7,7.6)	48.5 (23.9)	-6.2 (-12.9,0.5)	-10.8 (-20.5,-1.1)	0.03	8.1 (-2.6,18.8)	0.14	-10.6 (-21.3,0.1)	0.05

Variable	Low-income Pre-UIFSM	Difference	Mid-income Pre-UIFSM	Difference	High-income Pre-UIFSM	Difference	Low-income DiD[2]	P	Mid-income DiD[2]	P	High-Income DiD[2]	P
	Mean (SD)	Diff. (95% CI)[1]	Mean (SD)	Diff. (95% CI)[1]	Mean (SD)	Diff. (95% CI)	Coef. (95% CI)		Coef. (95% CI)		Coef. (95% CI)	
Control	38.5 (23.9)	2.1 (-5.3,9.4)	50.3[a] (32.2)	-8.5 (-17.7,0.7)	49.0b (24.6)	3.0 (-7.0,13.0)						
**Vitamin C (mg)**												
Intervention	20.4 (20.9)	1.0 (-6.9,8.9)	26.1 (20.6)	-6.5 (-14.2,1.2)	23.2 (20.6)	-5.9 (-11.9,0.1)	-4.1 (-14.6,6.3)	0.44	-3.1 (-13.4,7.3)	0.56	-5.1 (-14.6,4.3)	0.28
Control	21.3 (20.1)	4.0 (-3.7,11.8)	26.9 (23.5)	-3.8 (-11.9,4.3)	25.0 (21.1)	-0.9 (-9.0,7.2)						
**Vitamin A (ug)**												
Intervention	214.7 (252.1)	-47.1 (-122.5,28.3)	223.1 (324.6)	-67.7 (-147.5,12.1)	235.4 (239.7)	22.9 (-64.1,109.9)	-79.4 (-166.5,7.7)	0.07	-116.9 (-225.0,-8.8)	0.03	-14.3 (-133.4,104.9)	0.81
Control	126.7 (186.1)	15.0 (-28.5,58.5)	140.8 (154.8)	45.6 (-26.8,117.9)	168.5b (170.3)	26.7 (-44.8,98.1)						
**Fat (% of Energy)**												
Intervention	34.8 (9.6)	-2.8 (-5.8,0.2)	31.9 (8.0)	0.2 (-2.9,3.3)	33.0 (8.6)	-0.2 (-3.3,2.9)	-3.0 (-7.0,1.0)	0.14	-1.8 (-6.1,2.4)	0.39	-1.9 (-6.2,2.4)	0.38
Control	33.4 (8.3)	0.8 (-2.3,3.8)	32.6 (9.0)	2.1 (-1.0,5.2)	32.0 (8.2)	1.8 (-1.1,4.7)						
**Saturated Fat (% of Energy)**												
Intervention	12.9 (4.8)	-0.8 (-2.4,0.8)	11.6 (4.5)	0.0 (-1.9,1.9)	12.8 (5.0)	-0.8 (-2.5,0.9)	-1.7 (-4.0,0.5)	0.13	-0.3 (-2.7,2.2)	0.84	-0.4 (-2.5,1.7)	0.73
Control	11.4 (5.1)	1.0 (-0.6,2.6)	12.1 (4.6)	0.4 (-1.2,2.0)	11.7 (4.6)	-0.4 (-1.8,0.9)						
**Carbohydrate (% of Energy)**												
Intervention	52.3 (10.6)	3.5* (0.5,6.5)	55.3 (10.5)	0.6 (-3.2,4.4)	53.5 (10.3)	0.0 (-3.4,3.5)	3.8 (-0.3,7.9)	0.07	2.5 (-2.7,7.6)	0.34	1.7 (-3.3,6.8)	0.51

Variable	Low-income Pre-UIFSM	Difference	Mid-income Pre-UIFSM	Difference	High-income Pre-UIFSM	Difference	Low-income DiD[2]	P	Mid-income DiD[2]	P	High-Income DiD[2]	P
	Mean (SD)	Diff. (95% CI)[1]	Mean (SD)	Diff. (95% CI)[1]	Mean (SD)	Diff. (95% CI)	Coef. (95% CI)		Coef. (95% CI)		Coef. (95% CI)	
Control	55.3 (9.2)	-0.6 (-3.7,2.4)	56.1 (10.3)	-1.9 (-5.5,1.6)	55.5 (10.9)	-1.8 (-5.4,1.9)						
**NMES (% of Energy)**												
Intervention	12.1 (7.7)	0.3 (-3.0,3.6)	14.5 (9.3)	-2.8 (-5.7,0.2)	11.9 (8.7)	-1.8 (-4.1,0.6)	2.0 (-2.3,6.3)	0.36	-0.8 (-4.8,3.1)	0.69	-0.3 (-4.0,3.3)	0.85
Control	14.0 (9.3)	-1.3 (-4.4,1.8)	14.5 (9.3)	-2.3 (-5.2,0.6)	12.5 (7.8)	-1.4 (-4.4,1.6)						
**Protein (% of Energy)**												
Intervention	15.0 (4.4)	0.8 (-1.0,2.6)	15.2 (4.8)	0.2 (-1.3,1.8)	16.9b (7.7)	0.2 (-1.8,2.2)	0.4 (-1.9,2.8)	0.71	0.7 (-1.4,2.7)	0.53	-1.0 (-3.6,1.5)	0.44
Control	14.8 (4.3)	-0.1 (-1.7,1.5)	14.8 (4.5)	-0.3 (-1.7,1.1)	14.8 (4.6)	1.1 (-0.6,2.7)						

[1] The before-after difference tested using a survey adjusted t-test; [2] Linear regression adjusted for age, sex, ethnicity, household size, region, household income, IMD and total lunchtime intake (g). * P<0.05; a – significant difference between low-income and mid-income pre-UIFSM mean; b – significant difference between low-income and high-income pre-UIFSM mean Note: UIFSM -Universal Infant Free School Meal; DID – Difference-in-differences SD – standard deviation; Diff. – Difference; CI - confidence interval; Intervention – Infants (4-7 years); Control – Juniors (8-11 years) SD - standard deviation; CI - confidence interval; NMES – non-milk extrinsic sugar.
